# The temporal dynamics of emotion comparison depends on low-level attentional factors

**DOI:** 10.1038/s41598-023-33711-0

**Published:** 2023-05-05

**Authors:** Andrea Dissegna, Giulio Baldassi, Mauro Murgia, Francesco Darek Costa, Carlo Fantoni

**Affiliations:** Department of Life Sciences, University of Trieste, Via E. Weiss 21, 34128 Trieste, Italy

**Keywords:** Human behaviour, Attention

## Abstract

Humans are predisposed to attend to emotions conveyed by facial expressions. However, compulsory attraction to emotions gets challenging when multiple emotional stimuli compete for attention, as in the emotion comparison task. In this task, participants are asked to choose which of two simultaneously presented faces displays the most positive (happiest) or negative (angriest) emotion. Participants usually respond faster to the face displaying the most intense emotion. This effect is stronger for face pairs that contain globally positive rather than negative emotional faces. Both effects are consistent with an attentional capture phenomenon driven by the perceptual salience of facial expressions. In the present experiment, we studied the temporal dynamics of attentional capture in the emotion comparison task by tracking participants’ eye movements using gaze-contingent displays and responses. Our results show that, on the first fixation, participants were more accurate and dwelled longer on the left target face when it displayed the most intense emotion within the pair. On the second fixation, the pattern was reversed, with higher accuracy and longer gaze time on the right target face. Overall, our pattern of gazing behavior indicates that the typical results observed in the emotion comparison task arise from the optimal combination over time of two low-level attentional factors: the perceptual salience of emotional stimuli and the scanning habit of participants.

## Introduction

The ability to exhibit goal-directed behavior despite distractions is a crucial skill that requires conflict resolution through attentional control^1,2^. Attentional control refers to the capacity to regulate attentional focus, which can be affected by various factors such as low-level perceptual cues or high-level knowledge-based factors^3^. Emotion processing plays a relevant role in attentional control, as emotion and attention are inherently interconnected in real life situations^4^.

Here, we studied the process that regulates comparative judgments for optimal decision-making when multiple choices are available, as a measure of attentional control in emotion-directed behavior. Emotions are among the features for which attention can be involuntarily attracted, as they play a significant role in social communication and interaction^5,6^. However, recent evidence is contradictory, with reports of facilitation and impairment effects of emotion on the stimulus processing when the target stimulus conflict with distractors^7^. Moreover, several studies suggested that comparative judgements require high-level semantic and linguistic cognitive factors^8–11^. These high-level cognitive factors have been considered the *semantic* component of comparative judgment^8,12–16^. The influence of the semantic component on comparative judgement has been first supported by Banks et al.^8^. When participants were asked to choose the higher or the lower of two disks appearing as balloons tethered at the ends of strings, they were faster at choosing the highest of the two. The exact opposite pattern was observed when participants were instructed to interpret the same two disks as yo-yos hanging at the ends of strings. This pattern of choice resulted in a 2-way interaction between the type of task (higher vs. lower) and the type of instruction (balloons vs. yo-yos). This interaction was characterized by a funnel shape pattern of choice speeds with the speed of choice associated with one of the two types of tasks speeded up overall^8,17^. This further effect was attributed to a larger speed advantage for selecting the lower of two yo-yos, relative to the speed advantage for selecting the higher of two balloons.

Banks et al.^8^ interpreted the funnel-shaped pattern as the byproduct of a “semantic congruity effect” between a perceptual encoding of the stimulus and its semantic representation triggered by the instructions. The same stimulus was judged faster when placed in a spatial position congruent rather than incongruent with the knowledge of its typical position in the physical world, with balloons being associated with rising vertically, and yo-yos with falling vertically. Recent studies show that the funnel pattern emerges in various types of comparative tasks (direct vs. indirect^18^), stimuli related to different representational domains (symbolic vs. non-symbolic^19^), and, more importantly, in infants^20^ and monkeys^14^. The funnel pattern found in these studies cannot be explained by semantic knowledge alone.

## General framework

In the original seminal work by Banks et al.^8^, the authors proposed a model of the semantic congruity effect that incorporated a low-level perceptual stage in addition to the semantic component. This model account for the fact that the stimuli were analyzed at low-level perceptual stage before high-level processing. However, the relevance of the low-level perceptual stage is still a matter of debate.

Fantoni et al.^18,19,21^ observed that the standard pattern of data found in comparative judgment tasks was consistent with faster responses for the stimulus within the pair which is closest in terms of relative intensity to the extremal values of the series, regardless of semantic congruity. For instance, in the Banks et al.^8^ study, the stimulus within the pair with an extremal spatial position relative to the vertical (i.e. the *higher* of the two balloons or the *lower* of the two *yo-yos*) was faster regardless of the type of task. It is noteworthy that Fantoni et al.^18,19,21^ demonstrated the relevance of the low versus high-level stimulus properties using a direct emotion comparison task. In the direct comparison task, participants were asked to manually choose which of two simultaneously presented faces displayed the most positive (happiest) or negative (angriest) emotion. Participants were globally faster and more accurate in selecting the most intense emotion in the pair, regardless of the congruity between the spatial position of the faces (left vs. right) and any left-to-right mental representation of emotional valence (e.g. with the negative emotions being associated with the left side of the space and positive emotions being associated with the right side of the space^22–24^). In globally negative pairs (i.e. an angry|intermediate face pair), the most negative faces were selected faster and more accurately. Vice-versa, in globally positive pairs (i.e. a happy|intermediate face pair), the most positive faces were selected faster and more accurately. This result suggests that a Perceptual-Attentional Factor (PAF) alone may affect in a determinant way emotion comparison. According to this perspective, PAF is conceived as a stimulus-driven exogenous component of attention that generates an attentional capture effect, which is likely to be influenced by the perceptual salience of facial expressions. The effect in such a view depends on the prioritization in early sensory processing of salient (emotional happy/angry) over less salient (neutral) facial expressions of emotion^3,25–30^. Despite being independent of semantic factors, the pattern of results obtained by Fantoni et al.^18,19,21^ was fully consistent with the typical funnel pattern observed in the semantic congruity effect. For such a reason Fantoni et al.^18,19,21^ referred to their pattern as the “emotional semantic congruity effect”.

Differently from Banks et al.^8^, Fantoni et al.^18^ modelled the funnel pattern in the emotional domain as the direct speed-intensity association between motor reactivity/accuracy and the absolute intensity of the target emotion relative to the cut-off of a unidimensional continuum of emotion. This continuum represents anger as the negative pole and happiness as the positive pole, with 100 representing a fully emotional face and 0 representing a neutral face. Such a direct Speed-Intensity Association model (SIA) successfully predicted the funnel pattern in the domain of emotions providing an operationalization of the attentional capture effect produced by PAF^18^. When comparing stimuli with different perceptual salience or intensity, an attentional capture effect was observed. This effect slowed down motor reactivity and reduced choice accuracy when the task requires the selection of the intermediate rather than the extreme emotional face within a pair^19,31–36^. In addition, the attentional capture effect in emotion comparison task was consistent with two further effects that are typically observed in standard simultaneous comparison tasks. The two effects consist in the size effect, which refers to the increased speed and accuracy of response for globally positive (e.g., happy|intermediate) face pairs compared to negative (e.g. angry|intermediate) face pairs, and a happiness advantage, which refers to the emotion anisotropy that increases the speed and accuracy of choice for the happiest face rather than angriest face^36–40^. Both effects are predicted by SIA.

## Aim and expectations

Here, in order to unveil the role of low-level attentional factors in driving efficient comparative judgments in an emotion comparison task, we measured participants’ attentional shifts during different types of gazing tasks (gaze towards the happiest or angriest face within a pair). These tasks were conducted using the same horizontally oriented half emotional pairs used by Fantoni et al.^18^. These emotional pairs included real faces arranged side-by-side with opposite global valence but equal relative emotion intensity. Specifically, there were globally positive pairs, with an intermediate and happy facial expression (namely a 100|0 pair or 0|100), and*.* globally negative pairs, with an intermediate and an angry facial expression (namely a − 100|0 pair or 0|− 100). In contrast to previous emotion comparison tasks that measured speed and accuracy of choice using bimanual responses, we used gaze as a probe of choice accuracy. Tracking the eyes during the comparison task allowed us to measure the accuracy of the choice of the target face in terms of fixation accuracy (e.g. the probability of casting the gaze on the region of interest covering the target face) and dwell time (e.g. the total amount of time the gaze is cast on the region of interest covering the target face) depending on the specific gazing instruction. Both indices of gaze-contingent choice behavior were treated as windows on low-level attentional processes.

In particular, our indices of gaze-contingent choice behavior are relevant in addressing an unresolved question from Fantoni et al.’s^18,21^ study. This question regards the different funnel patterns in spatially congruent and incongruent conditions. Authors found that selecting faces in spatially congruent emotional pairs (namely pairs displaying the happy face to the right and the neutral to the left or the angry face to the left and the neutral to the right) led to a reduction of the funnel pattern. The reduction of the funnel pattern was found to be due to a smaller happiness advantage compared to selecting faces in spatially incongruent emotional pairs (namely pairs displaying the happy face to the left and the neutral to the right or the angry face to the right and the neutral to the left). Throughout the paper, we refer to the difference between the funnel pattern in spatially congruent and incongruent conditions as the *spatial congruity anisotropy*.

Importantly, in Experiment 2 of Fantoni et al.^18^ the spatial congruity anisotropy was found to be particularly evident when emotional pairs were displayed tachistoscopically rather than until participant response. The reason for such a difference remained unsolved, although authors hypothesized that such a difference resulted from an endogenous factor of attention modulating PAF^41–50^. This endogenous factor of attention might be driven by the scanning habit, that prioritizes stimuli either on the left or on the right visual hemifield depending on the temporal phase of the scanning of the visual scene. We will refer to this second attentional factor as the Automatic-Attentional Factor (AAF). Previous research has shown that initial fixations toward faces are biased toward the left side of the face, followed by a rightward fixation^43,48^. Whether this scanning habit bias is related to innate or cultural factors, like the reading direction, is still a matter of debate.

It is possible that the tachistoscopic view of a lateralized pair of emotions forced the attentional system to focus on the first (e.g. the left) hemifield rather than on the second (e.g. right) hemifield as an effect of a leftward bias produced by AAF. It was likely that the direction of such a bias was supported by cultural factors being the sample of participants tested by Fantoni et al. (Experiment 2)^18^ selected from western culture and exposed to left-to-right reading/writing direction. This would explain the overall speeding up/larger accuracy of the selection of left-emotional rather than right-emotional targets found by Fantoni et al. (Experiment 2)^18^. In congruent spatial conditions, participants selected the angriest face (on the left) of an emotional pair faster and more accurately than the happiest face (on the right). Conversely, in incongruent spatial conditions, participants selected the happiest (on the left) face of the emotional pair faster and more accurately rather than the angriest (on the right) face.

In the present study, we hypothesized and tested whether the spatial congruity anisotropy is due to the combination, over exploration time of a face pair, of PAF (predicting a funnel pattern which is similar over spatial congruency conditions) and AAF (predicting a left or right hemifield advantage depending on the spatial congruency condition over exploration time). The actual involvement of these factors in comparison tasks has never been directly demonstrated, despite previous studies indicated that the combination of these factors may result in the funnel pattern of emotion comparison tasks^18,19,21^.

A revised version of SIA that integrates PAF and AAF (integrated SIA) can predict the different funnel patterns over spatial congruency conditions (i.e. the spatial congruity anisotropy) as follows. If, according to AAF, fixations are more likely to occur on the left visual hemifield rather than on the right, the intensity of targets on the left will be weighted more heavily compared to the intensity of targets on the right, producing a *left-hemifield advantage*. This left-hemifield advantage modifies the funnel pattern predicted by PAF alone as in the standard SIA that will be differently shaped depending on the spatial congruency conditions. In particular, the lines joining the right or left fixation accuracy over the Average Emotion Intensity of the pair are predicted to cross over at different points depending on spatial congruency. The crosspoint in the average emotion intensity continuum will be shifted towards negative values for spatially incongruent pairs, and towards positive values for spatially congruent pairs. According to the categorization of emotion comparison effects proposed by Fantoni et al.^18^, a shift in the negative direction produces a happiness advantage. This type of advantage involves a larger difference between fixation accuracy for fully and intermediate emotional faces in globally positive rather than globally negative emotional pairs. Notably, the exact opposite unbalance between funnel patterns over spatial congruency conditions is expected to occur in the case of a *right-hemifield advantage*, when fixations are more likely to occur on the right rather than on the left visual hemifield.

In the present study, to test the validity of the integrated SIA model, we conducted an experiment on a sample similar to Fantoni et al.^18^ (Italian native speakers with left-to-right reading/writing direction), using a gazing instead of bimanual response. We analyzed the global fixation accuracy, dwell time and saccade latency and investigated the temporal dynamics of gazing performance. We analyzed eye-movements at the beginning and at the end of the gazing task, expecting different left and right hemifield advantages (produced by the scanning habit) in each temporal period.

## Experiment

How does the deployment of attention, reflected by eye-movement patterns during a gaze-contingent emotion comparison task, differ over time depending on the engagement of PAF versus AAF?

In order to answer this question, we focused on three attentional indices of gaze-contingent choice behavior: the fixation accuracy, dwell times, and the saccade latency. These indices were extracted from the individuals’ flow of eye movements performed during an emotion comparison task. They were measured among 8 types of half-range emotional pairs, resulting from the factorial combination of 2 Target Position (Left vs. Right) × 2 Spatial Congruency with the mental spatial representation of valence (Congruent if the happiest face is displayed on the right hemifield, and Incongruent if the happiest face is displayed on the left hemifield) × Average Emotion Intensity (50 for happy|intermediate and − 50 for angry|intermediate pairs).

The assumption of our paradigm is that attentional indices of gaze choice mirror response accuracy and speed in the bimanual version of standard emotion comparison tasks. SIA predictions^11^ and its integrated version (see subsection “Aim and expectations”) should thus account for our pattern of eye movements as well. As a consequence, we expect the pattern of attentional indices to be characterized by the following effects:

1. A crossover resulting from a 3-way Average Emotion Intensity × Target Position × Spatial Congruency interaction. This interaction is diagnostic of an effect of PAF alone, with the lines connecting fixation accuracy/dwell time for the left and right targets crossing when plotted against average emotion intensity in both congruent and incongruent spatial positions;
2. A main effect of the Average Emotion Intensity. This effect is diagnostic of a size effect, which involves a global shearing of the crossover pattern in (1) with the fixation accuracy/dwell time for globally negative pairs being smaller than the fixation accuracy for globally positive pairs;
3. A 2-ways Spatial Congruency × Target Position interaction. This interaction is diagnostic of an emotion anisotropy compatible with either a happiness (with a fixation accuracy/dwell time advantage for happy target faces in right congruent or left incongruent spatial position better than angry faces in right congruent or left incongruent spatial position) or an anger advantage (with a fixation accuracy/dwell time advantage for angry target faces in right congruent or left incongruent spatial position better than happy faces in right congruent or left incongruent spatial position).

If the emotion anisotropy in (3) is present, then the pattern of choice in (1) is turned into a funnel-shaped pattern. This type of pattern is diagnosed by a 3-way interaction with a shift of the cross point between the lines connecting the fixation accuracy/dwell time for left vs. right targets towards negative (in the case of a happiness advantage) or positive (in the case of an anger advantage) values of average emotion intensity. Furthermore the 2-ways interaction in 3) can be nulled in the presence of a significant spatial congruity anisotropy. If the funnel pattern in congruent and incongruent spatial positions are similar in magnitude but opposite in direction (i.e. with one funnel pattern compatible with a happiness and the other with an anger advantage), than the 2-way interaction will indeed be nullified. This result is expected if AAF (beyond PAF) is at work.

In order to test how these expected set of effects changes over gazing time, we capitalized on a novel approach introduced by Schurgin et al.^48^. In their experiment Schurgin et al.^48^, focused on a limited set of informative fixations to explore the time course of fixation accuracy and dwell time during the exploration of different types of facial expressions. They showed that the first and second fixations are the most variable across time and conditions. Authors thus concluded that the first and second fixations are the most informative in terms of fixation accuracy/dwell time. This is also consistent with results by Caspi et al.^51^ (but see also^52–54^) showing that most of the information that guides the eye movement in a visual search task is accumulated within the first 200 ms of the display presentation. Additionally, the first fixations and saccades are typically dependent on perceptual salience, which is either value-driven or stimulus-driven. According to these findings, we focused our analysis on the temporal dynamics of gazing measuring the first and the second fixation accuracy and dwell time (the selection of these two fixations resulted to be the most informative as corroborated also by the results of the preliminary analysis in the “Data analysis” subsection).

## Methods

### Participants

Participants gave informed consent prior to inclusion in the study. They were treated in compliance with national legislation, the Ethical Code of the Italian Association of Psychology (approval of the Research Ethics Committee of the University of Trieste number 84c/2017), and the Code of Ethical Principles for Medical Research Involving Human Subjects of the World Medical Association (Declaration of Helsinki).

Thirty-three participants obtained course credits to take part in the experiment. They were Italian speakers (i.e. left-to-right reading direction), naïve to the purpose of the study, and had normal/corrected-to-normal visual acuity. Participants were 29 females and 4 males (average age = 21.88 ± 2.88 SD; age range = [19–33]). Their handedness was measured with the 10-item Edinburgh Handedness Inventory^55^. The average quotient of lateralization of 43.47 (SD =  ± 49.70; min. to max. range = [− 85–100]), revealing that, on average, our participants were right-handed.

We conducted a sensitivity analysis with G-Power 3.1^56^ on our sample size with α err. Prob. = 0.05, Power (1—β err. Prob.) = 0.90 to establish the Minimal Detectable Effects resulting from our experimental design. These resulted to be in the medium-to-large range with a critical *F* = 2.02 and a η_p_^2^ = 0.10.

### Material

Face pairs were presented on a 23.6-inch. ViewSonic color monitor (1920, 1080, 60 Hz) and generated with a custom-made program written in Experiment Builder (SR-Research, Ontario, Canada) running on a Dell Precision T3500 machine (Windows 7 Ultimate). From the participant's point of view (58 cm far from the monitor), each face measured 6.92° height and 5.46° wide. The eccentricity of each face within a pair (nose-to-medial axis of the screen) was 10.92°. According to the results of Bayle et al.^57^, the eccentricity of our stimuli guaranteed a lateralized encoding of each face and was within the limits of the human ability to detect facial expressions. This is also consistent with results by Fantoni et al.^18,19^ showing that the exact same pairs of facial expressions displayed at similar eccentricity led to accurate emotion comparison performance in a bimanual task.

We used the same colored-photographs of human facial expressions used by Fantoni et al.^18,19^. The photographs of faces were taken from 8 Caucasian Characters (4 female and 4 male) selected from the Radboud University Nijmegen set^58^ (Character numbers: 1, 2, 4, 19, 20, 30, 46, and 71). The hair and ears of the faces were masked by a black oval vignette^18^. For each Character, we obtained a set of 3 facial stimuli belonging to the anger-to-intermediate-to-happiness continuum. The intermediate face of each Character (emotion intensity = 0) was paired with his/her fully emotional face (Angry: emotion intensity = − 100; Happy: emotion Intensity =  + 100). As a result, we obtained 4 types of half-range emotional pairs defined by 2 Spatial Congruency × 2 Average Emotion Intensity relative to the cutoff intermediate face (globally negative pairs: Average Emotion Intensity = − 50; globally positive pairs: Average Emotion Intensity =  + 50) conditions. The spatial position of the target face could either be on the right or on the left-hemifield thus defining two further levels of analysis (Target Position = Left/Right).

Eye movements were recorded with an EyeLink 1000 Desktop Mount system (sampling rate: 1000 Hz; Average gaze position accuracy of 0.15°. SR Research, Ontario, Canada) in a head-free tracking setting. The recording was from the left eye, though viewing was binocular. A standard 13-point calibration phase was performed before the beginning of each experimental block.

### Experimental design

The experimental design included 64 trials per block. The trials resulted from the full factorial combination of 8 Types of Character × 2 Target Position × 2 Spatial Congruency × 2 Average Emotion Intensity. Within each block, participants performed the same task but following different instructions: (A) “gaze towards the angriest face within the pair” or (B) “gaze towards the happiest face within the pair”. The ordering of the instruction block was fully counterbalanced across participants with half of the participants performing the experiment following the A-B and half the B-A order.

### Procedure

The procedure was similar to the one adopted by Fantoni et al.^18,19^, but with the aid of gaze-contingent control of choice behavior. Participants were asked to show up at the lab with no eye makeup to guarantee a precise and accurate parsing of gazing by the Eye Tracker. On their arrival, participants underwent the Edinburgh Handedness Inventory and received general oral instructions about the experiment. They sat in a dimly illuminated, acoustically isolated room. The experiment was introduced by on-screen instructions (“gaze towards the angriest/happiest,” depending on instruction order). On-screen instructions informed participants that they had to choose among a pair of horizontally aligned faces which of the two appear to be the angriest/happiest, moving their gaze on the target face. They were also instructed to control the advancement of the trial with the spacebar while keeping their position relative to the screen stable during the experiment. After carefully setting the participant hand and body position, the experimental block began with the eye-tracking calibration phase. This calibration phase was repeated, as needed, throughout the experiment. After eye calibration, the trial started with a gaze-contingent fixation phase in which a red cross appeared at the center of the monitor. If the gaze was outside the circular fixation region (radius = 1°) surrounding the cross for more than 15 s, the task was momentarily interrupted, and the participant enters again the eye calibration phase. If the gaze was inside the fixation region, the red fixation cross then turned into a green cross, indicating that the participant could proceed to the response phase by pressing the space bar on the keyboard. After the space bar press, the fixation cross turned white and remained on view for a variable [800–1300 ms] time interval. This variable time interval is used to prevent any anticipatory saccadic movement^59^. The fixation cross was then replaced by the face pair display. The observer was required to cast the gaze on either the happiest or angriest face within the pair (depending on the Instruction condition). The stimulus remained on screen for 3000 ms after the first fixation was detected by the eye-tracker over one of the two regions of interest surrounding each face (ROIs, 7.92° height and 6.46° wide). This exploration phase was intended to provide the participant enough time to freely explore the face pair or to correct the first gaze choice behavior. After this phase, the stimulus was replaced by a 3000 ms blank refresh screen.

### Data analysis

The EyeLink online parser uses a “saccade picking” approach, based on instantaneous velocity and acceleration thresholds to determine the onset and offset of saccades. Samples above the thresholds are determined to be in saccade, and samples below the thresholds are determined to be in fixation. The parser setting uses velocity and acceleration thresholds of 30°/s and 8000°/s^2^, respectively. So, the onset of a fixation is determined by the offset of the previous saccade, and the offset of a fixation is determined by the onset of the subsequent saccade. Fixations cast within or between each oval region of interest matching each face of the pair were detected as long as there was no saccade in progress. We analyzed data from 9254 individual fixations. Each individual fixation was encoded in terms of its dwell time (ms), its fixation accuracy (1 = the fixation is cast on the oval region of interest surrounding the target face; 0 = the fixation is cast on oval region of interest surrounding the face that was not the target), and its order (whether the 1st, 2nd and so on). Descriptive statistics of fixations is reported in the Supplementary Materials (subsections 1.1, 2.1 and 3.1).

Following Schurgin et al.^48^ (but see also Caspi et al.^51^) in a preliminary analysis of fixation accuracy, we determined the number of informative fixations in our task in order to restrict the successive analyses to informative fixations and saccades only. The analysis showed that fixation accuracy reached a plateau (90%, SD = 0.29) on the third fixation. The performance was fully stabilized across time and conditions on the fourth fixation (*F*(1, 3376) = 3.83, *p* = 0.051). The fixation accuracy of the third fixation was at ceiling over the experimental conditions revealing a null 3-way Average Emotion Intensity × Target Position × Spatial Congruency interaction. Hence, in the subsequent part of the paper we focused the analysis on the most informative fixations and saccades only: the first and the second^48^ for a total of 4133 fixations and their associated saccades. From the individual saccades associated with these fixations, we extracted the latency and type. The type of saccades was obtained by encoding them as either corrective, when occurring between-faces, or confirmatory, when occurring within the same face. Descriptive statistics of saccades are reported in the Supplementary Materials (subsection 3.1). As a further measure of gazing behavior, we analyzed dwell times (see the subsection 2.1 of the Supplementary Material for descriptive statistics).

Following Barr et al.^60^, we performed our analyses using generalized linear mixed models with the maximal random effect’s structure justified by our experimental design: These consisted in a MAX*glmer* (with probit as the link function for fixation accuracy) and a MAX*lmer (*for dwell time and saccade latencies), with a by-subject slope and intercept for each condition resulting by our Average Emotion Intensity × Target Position × Spatial Congruency design and the full Emotion Intensity × Target Position × Spatial Congruency as fixed factor. Our MAX*lmer* and MAX*glmer* estimates, and *p*-values are reported in the Supplementary Material (see subsection 1.2 for fixation accuracy, 2.4 for dwell times and 3.3 for saccade latencies). We performed Welch’s *t*-Test to contrast the conditions involved in our interactions. To examine the time course of gazing behavior, we conducted an analysis including the Fixation count (1 for first, 2 for second) as a further factor in the Average Emotion Intensity × Target Position × Spatial Congruency MAX*glmer/* MAX*lmer* model.

The analysis of dwell times can be found in the Supplementary Materials of the manuscript (see Section 2). The decision to exclude it from the main text was due to considerations of brevity, and because the analysis produced results that were similar to those of fixation accuracy. For similar reasons, the analysis of saccade latencies was not reported in the manuscript in its extended form.A preliminary analysis revealed that the distributions of individual first and second saccade latencies were fully accounted for by their associated fixation accuracies. This was demonstrated by a MAX*lmer* (*r*_c_ = 0.31, 95% CI [0.29, 0.32], see Table 9 Supplementary Materials) including the fixation accuracy as an additional covariate of saccade latency beyond each condition resulting from our Average Emotion Intensity × Target Position × Spatial Congruency design (*F*(1, 31.88) = 2.04, *p* < 0.001). Fixation accuracy nulled the Target Position × Spatial Congruency × Average Emotion Intensity interaction (*F*(1, 153.23) = 0.014, *p* = 0.903) showing that there was no evidence that the pattern of saccades latency was accounted for by any factor beyond fixation accuracy. Overall, there was a positive relationship between saccade latency and fixation accuracy, with longer saccades associated with larger likelihood of fixation accuracy (*β* = 138.25, s.e. = 43.72, *t*(72.10) = 3.16, *p* = 0.002).

As a final analysis, we tested the predictive power of the integrated SIA model on fixation accuracy and dwell times. In doing that, we followed the procedure proposed by Fantoni et al.^18^. We predicted the pattern of fixation accuracy/dwell time with the linear combination of emotion intensity values that models the PAF and produce the crossover, the size effect and the emotion anisotropy effect typically observed in emotion comparison. Furthermore, we integrated the SIA adding the AAF based on the scanning habit component as an additional parameter. In particular, the AAF was modelled as a weight modulating the emotion anisotropy component of standard SIA depending on the Spatial Congruency conditions. In order to do that, individuals’ fixations accuracy and dwell times were used to first extract 2 individual synthetic indexes of emotion anisotropy, one for each condition resulting from the combination of 2 Target Position × 2 Spatial Congruency conditions. Each individual index of emotion anisotropy was calculated by subtracting the individual value of the best fitting MAX*glmer* (on fixation accuracy)/MAX*lmer* (on dwell time) regressor’s intercept for the selection of a target on the Left *vs* Right Target Position in Congruent and Incongruent Spatial Positions over Average Emotion Intensity. These regressors correspond to the line connecting the fixation accuracy/dwell time for a target on the Left (i.e. the angriest|emotional face and the angriest|intermediate face in spatially congruent pairs, and the happiest|intermediate face and the happiest|emotional face in spatially incongruent pairs) and the line connecting the fixation accuracy/dwell time for a target on the Right (i.e. the happiest|intermediate face and the happiest|emotional face in spatially congruent pairs, and the angriest|intermediate face and the angriest|emotional face in spatially incongruent pairs). When applied to each Congruency condition, this procedure leads to the identification of two values of emotion anisotropy: one for the congruent (*k*_cong_) and one for the incongruent (*k*_incong_) condition. In both cases, these indices quantify how much an individual fixation is biased by the selection of the most positive *vs.* negative emotion within the pair, with positive values indicating a shift of the Crosspoint between the Left vs. Right lines towards negative values of Average Emotion Intensity. This is consistent with a funnel pattern, producing a *happiness advantage* (i.e., an unbalance in favor of the selection of the most positive face when the average emotion intensity is null). Negative values of the *k* index, instead, indicate that the Crosspoint of the two lines is biased towards positive values of Average Emotion Intensity. This bias is consistent with a funnel involving an *anger advantage*. In our analysis, the difference between individuals’ *k*_cong_ and *k*_incong_ is revealed through Welch t-Test and interpreted as diagnostic of the spatial congruity anisotropy.

In addition, we calculated the individual values diagnostic of AAF modelling the scanning habit (*c*) and integrated such a parameter into the SIA. In order to do that, we considered that *k*_cong_ and *k*_incong_ resulted from a linear combination of the global emotion anisotropy (*K,* as a synthetic value of the emotion anisotropy in congruent and incongruent conditions) and *c,* representing the effect of scanning habit on *k*_cong_ and *k*_incong_, (with *c* exerting opposite effects on *k*_cong_ and *k*_incong_) as expected by AAF. Based on this line of reasoning, we obtained *K* and* c* by solving the following system of equations for each individual value of *k*_*cong*_ and* k*_*incong*_:

1 $$\left\{\begin{array}{c}{k}_{cong }=K+c\\ {k}_{incong}=K-c\end{array}\right.$$

From the previous system of equations, we computed individuals’ values of *c* as follows:

2 $$\mathrm{c}= \frac{{k}_{cong}-{k}_{incong}}{2}$$

According to Eqs. (1) and (2), a negative value of *c* represents a left-hemifield advantage with *k*_incong_ > *k*_cong_. When *c* is negative *k*_cong_ is biased towards negative values in the direction of an anger advantage, relative to a condition in which the spatial congruity anisotropy is null (i.e. with *c* = 0 and *K* = *k*_incong_ = *k*_cong_). The opposite occurs to *k*_incong,_ which is biased towards positive values in the direction of a happiness advantage. A positive value of *c* represents a right-hemifield advantage, as it results from a *k*_cong_ which is larger than *k*_incong_. The way the parameter *c* varies over time captures the individual's scanning habit, without making any prior assumptions about the direction of that habit (being it left-to-right or right-to-left).

As a synthetic measure of the balance between PAF and AAF, we analyzed individuals’ Michelson contrast (*M*_*c*_) of* c*—*K* with *M*_*c*_ ranging from − 1 to + 1. Negative values indicate an unbalance in favor of PAF over AAF, while positive values indicate the opposite. In particular, we characterized every individual pattern of spatial congruity anisotropy between congruent and incongruent conditions as a specific combination of PAF (predicting a fully symmetric crossover with *c* = 0) and AAF (predicting an overall advantage of the performance when the target is in one hemifield rather than the other with *c* ≠ 0). We tested the unbalance between the PAF and AAF by means of one sample *t*-Test of individual *M*_*c*_ vs. 0.

We quantitatively tested the accuracy of our SIA-based predictions by remapping the entire set of individual values associated with our experimental factors and applying the linear combination of intensity components predicted by the integrated SIA. This meant recoding each individual target face value within a pair in terms of the sum between the following factors:

1. The Absolute Emotional Intensity, which was given a value100 for angry and happy, and 0 for intermediate faces;
2. A per-subject weighed empirically determined value (α) modeling the size effect. This value was extracted following Fantoni et al.^18^ as the best individual fitting multiplying factor of the Average Emotion Intensity (+ 50 for globally positive pairs and − 50 for globally negative pairs);
3. A per-subject empirically determined value of emotion anisotropy (*K*);
4. A per-subject empirically determined value of scanning habit (*c*).

To test whether SIA remapped values predicted the pattern of fixation accuracy/dwell time, we performed a MAX*glmer/*MAX*lmer* analysis testing the effects of Spatial Congruency and Target Position, including the SIA remapped values as a mediator. We also provided the *R*^2^ for the fitting between SIA based predicted and observed values.

As statistical inferential measures, we provided: (1) type III-like two-tailed *p*-values for significance estimates of MAX*glmer* and MAX*lmer* fixed effects and parameters adjusting for the F-tests the denominator degrees of freedom with the Satterthwaite approximation; (2) estimates of effect size based on the concordance correlation coefficient *r*_*c*_, Partial eta squared η_p_^2^ (for the interactions and main effects of the *F*-tests), and Cohen’s *d* (for the Welch’s two sample *t*-Tests).

Analyses were performed using the R-4.2.2 package lme4 (version 1.1–31). Model fits derived from MAX*glmer* and MAX*lmer* parameter estimates were provided in the Supplementary Materials.

### Ethical approval

The present study was approved by the Research Ethics Committee of the University of Trieste in compliance with national legislation (Approval Number 84c/2017), the Ethical Code of the Italian Association of Psychology and the ethical standards laid down in the 1964 Declaration of Helsinki and its later amendments. Participants gave informed consent prior to inclusion in the study.

## Results

### Fixation accuracy

**Figure 1:**
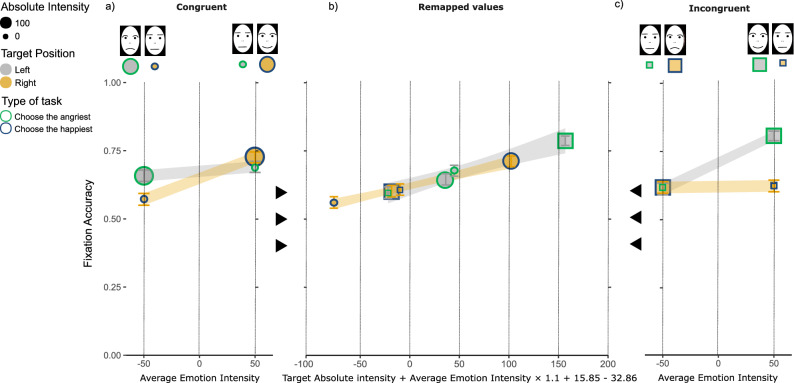
Distribution of fixation accuracy calculated over the first two fixations. (**a–c**), illustration of the mean fixation accuracy in Spatially Congruent (circles in panel (**a**) and Spatially Incongruent (squares in panel (**c**) conditions. Within each panel, mean fixation accuracies are shown as a function of Average Emotion Intensity (panels (**a**) and (**c**) and as a function of the best SIA remapped values (panel (**b**)). SIA remapped values of experimental condition intensities were obtained including the 3 empirically determined values modelling: (1) the size effect, (2) the emotion anisotropy and the (3) the scanning habit (equation below the *x-axis* from the leftmost to the rightmost free parameter). Error bars represent ± 1 s.e.m. and the size of the circles represent the absolute emotion intensity (small = intermediate; large = 100 per cent angry or 100 per cent happy). The Target Position and the type of task are coded by the colors filling and bounding the circles, respectively (legend). Gray/yellow lines in panels a-c are the best fitting MAX*glmer* regression lines for Left/Right Target Position conditions, with the shaded bands corresponding to ± 1 standard error of the regression. On the top of panels a and c, the schematic representations of the photographs of human facial expressions that we used in the experiment (for more details see the “Material” subsection).

Figure 1 depicts participants’ fixation accuracy pooled across the first two fixations. The pattern of data closely matches to the pattern of motor reactivity and accuracy found by Fantoni et al.^18^ in the standard bimanual version of the emotion comparison task.

In Fig. 1, the distribution of fixations accuracy is characterized by a funnel pattern with best fitting *glmer* regression lines connecting fixation accuracy for left (grey lines) and right (yellow lines) targets crossing over in a true interaction when plotted against Average Emotion Intensity. This occurs in both congruent (panel a) and incongruent (panel c) spatial positions. However, the pattern of fixation accuracy reveals a rather evident spatial congruity anisotropy with the funnel pattern in spatially congruent conditions compatible with an anger advantage (*glmer* regressors crossing for negative values of average emotion intensity) and the funnel pattern in spatially incongruent conditions compatible with a happiness advantage (*glmer* regressors crossing for negative values of average emotion intensity). Despite this difference, the funnel pattern observed in both congruent and incongruent conditions can be fully explained by the values of individuals’ fixation accuracy, which were remapped according to the most parsimonious linear combination of intensity components predicted by an integrated SIA (Fig. 1, panel b: empirically determined free parameters: *K* = 15.85,* c* = − 32.86, and *α* = 1.1). The fact that the optimal fitting values of *K* and* c* resulted to be non-null suggests that both PAF and AAF are engaged in the shaping of the pattern of fixation accuracy producing a funnel shape, with the non-null value of* c* standing for a spatial congruity anisotropy.

These observations were corroborated by a MAX*glmer* model (*r*_c_ = 0.19, 95% CI [0.17, 0.20], see Table 1 Supplementary Materials). The MAX*glmer* analysis revealed:

1. An Average Emotion Intensity × Target Position × Spatial Congruency interaction, *F*(1, 4088) = 33.49, *p* < 0.001, η_p_^2^ = 0.424, 95% CI [0.206, 0.585]. This was consistent with a globally consistent with a funnel-shaped interaction in both Congruent and Incongruent Spatial Position conditions.
2. a main effect of Average Emotion Intensity, *F*(1, 26) = 36.78, *p* < 0.001, η_p_^2^ = 0.466, 95% CI [0.230, 0.604]. This was consistent with a size effect in the domain of emotions with participants' fixation accuracy increased from globally negative to globally positive face pairs with a rate of *β* = 0.091 ± 0.086, *t*(4116.7) = 2.29, *p* < *0.0*01, *d* = 2.29.

No other main effect or interaction was significant (*p* > 0.06), including the Spatial Congruency × Target Position interaction (*F*(2.90, 4088) = 2.90, *p* = 0.080) diagnostic of an emotion anisotropy.

According to our expectations, the funnel pattern diagnosed by the significant 3-ways interaction in the absence of a Spatial Congruency × Target Position was due to a left-hemifield advantage (*c* = − 32.06 ± 17.67, *t*(32) = − 1.81, *p* = *0.0*40; *d* = − 0.32, 95% CI [− 0.67, 0.04]), and a happiness advantage (*K* = 15.85 ± 8.02, *t*(32) = 1.97, *p* = *0.0*29; *d* = 0.34, 95% CI [− 0.01, 0.70]). The perceptual and automatic attentional factors were unbalanced in favor of AAF over PAF as signaled by *M*_*c*_ = 0.28 (95% CI [0.07, 0.44], *t*(32) = 2.80, *p* = *0.0*09; *d* = 0.49, 95% CI [0.12, 0.86]). According to our hypothesis, this unbalance indicated a spatial congruity anisotropy, which was further corroborated by the analysis of the crosspoint between best fitting *glmer* in congruent *k*_cong_ and incongruent spatial position *k*_incong_.

In spatially congruent pairs, the *k*_cong_ was negative as consistent with a funnel pattern compatible with an anger advantage (*k*_cong_ = − 16.24 ± 24.52, *t*(32) = − 0.66, *p* = 0.256; *d* = − 0.12, 95% CI [− 0.46, 0.23]).The opposite occurred in spatially incongruent pairs in which the *k*_incong_ was positive as consistent with funnel pattern compatible with a happiness advantage (*k*_incong_ = 47.89 ± 12.34, *t*(32) = 3.88, *p* < 0.001; *d* = 0.68, 95% CI [0.30, 1.07]). A significant difference between the *k*_cong_ vs *k*_incong_ (difference = 64.13, 95% CI [8.90, 119.36], *t*(47.24) = 2.34, *p* = 0.024; *d* = 0.58, 95% CI [0.08, 1.07]) was also observed as again supporting a spatial congruity anisotropy.

Additional evidence of the spatial congruity anisotropy results from the post-hoc analysis on average fixation accuracies. The post-hoc analysis showed a large advantage of emotional over intermediate faces in spatially congruent pairs with a negative Average Emotion Intensity (M_angriest|emotional_ = 0.65 ± 0.02, vs. M_happiest|intermediate_ = 0.57 ± 0.02, *χ*^*2*^(1) = 3.51, *p* = 0.061,* d* = 0.69). This large advantage was opposed to the small advantage of emotional over intermediate faces in spatially congruent pairs with positive Average Emotion Intensity (M_angriest| intermediate_ = 0.69 ± 0.02, vs. M_happiest|emotional_ = 0.72 ± 0.02, *χ*^*2*^(1) = 1.08, *p* = 0.298, *d* = 0.37). The opposite pattern was observed in spatially incongruent pairs. In this condition, we observed a large advantage of emotional over intermediate faces for positive Average Emotion Intensity (M_angriest| intermediate_ = 0.62 ± 0.02, vs. M_happiest|emotional_ = 0.80 ± 0.02, *χ*^*2*^(1) = 18.82, *p* < 0.001,* d* = 2.30) but not for negative Average Emotion Intensity (M_angriest|emotional_ = 0.61 ± 0.02, vs. M_happiest| intermediate_ = 0.61 ± 0.02, *χ*^*2*^(1) = 0.02, *p* = 0.882,* d* = 0.05).

As a final analysis, we quantitatively tested the goodness of the integrated SIA predictions (details in Section “Data analysis”). A MAX*glmer* including the SIA remapped values as an additional covariate (with empirically determined parameters* K* = 15.85,* c* = − 32.86 e α = 1.1), revealed that SIA was the only significant factor (*F*(1, 4088) = 70.26, *p* < 0.001, η_p_^2^ = 0.615, 95% CI [0.416, 0.724]) nulling the Target Position × Spatial Congruency × Average Emotion Intensity interaction (*F*(1, 4088) = 0.254, *p* = 0.613). In conclusion, we found no evidence that the pattern of pooled fixation accuracy was accounted for by any factor beyond SIA remapped values.

### Fixation accuracy over time

**Figure 2:**
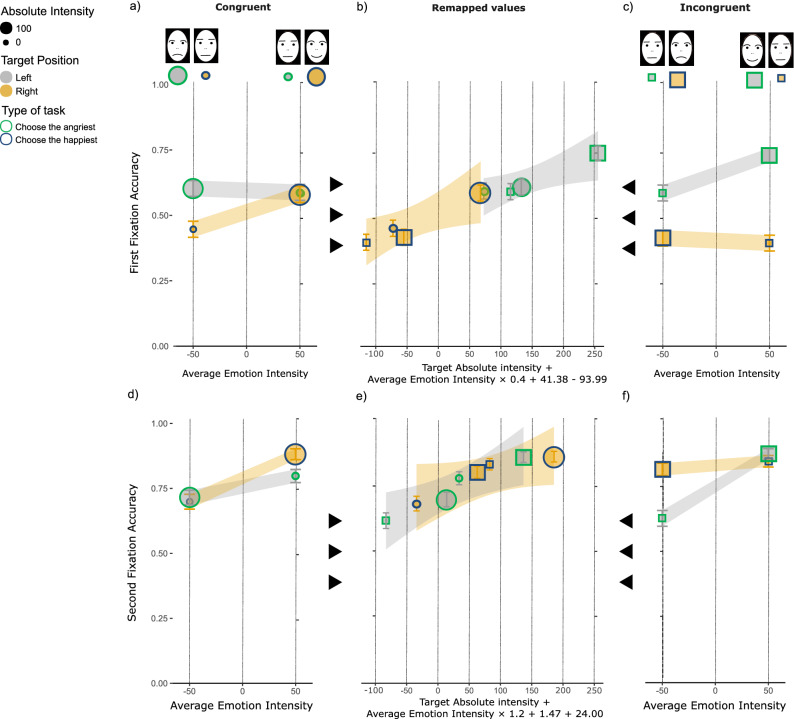
Distribution of First and second fixation accuracy. (**a–c**) depiction of the mean of the first and second fixation accuracy (**d–f**) in Spatially Congruent (panel (**a**) and (**d**)) and Spatially Incongruent (panel (**c**) and (**f**)) conditions. Within each panel, mean fixation accuracies are shown as a function of Average Emotion Intensity (panels a, c, d and f) and as a function of the best SIA remapped values (panels (**b**) and (**e**)). SIA remapped values of experimental conditions intensities were obtained including the 3 empirically determined values modelling: (1) the size effect, (2) the emotion anisotropy and the (3) the scanning habit (equation below the *x-axes* from the leftmost to the rightmost free parameter). The same symbols and color encoding of variables used in Fig. 1 is used as by the legend on top with error bars indicating ± 1 standard error of the mean and gray/yellow lines in panels a-f are the best fitting MAXg*lmer* regression lines for Left/Right Target Position conditions, with the shaded bands corresponding to ± 1 standard error of the regression. On the top of panels a and c, the schematic representations of the photographs of human facial expressions that we used in the experiment (for more details see the “Material” subsection).

Following Schurgin et al.^48^, we addressed the origin of the different funnel patterns in spatially congruent vs. incongruent pairs, analyzing the temporal dynamics of fixation accuracy in the First and Second fixation separately. This analysis is meant to validate the expectation that AAF regulates the gazing behavior over time producing an opposite pattern of spatial congruity anisotropy in the First and Second fixations. The pattern of data depicted in Fig. 2 corroborated such an expectation. Figure 2 shows the distribution of the First (panels a–c) and Second (panels d–f) fixation accuracy as a function of Average Emotion Intensity for Spatially Congruent (panel a–d) and Incongruent pairs (panel c–f). The same distribution of fixation accuracy was plotted as a function of the integrated SIA remapped values in panels (b) and (e). Notably, the funnel pattern observed in the First fixation (Fig. 2, panels a–c) is different from the one observed in the Second fixation (Fig. 2, panels d–f). The accuracy of the first fixation is characterized by a funnel pattern compatible with an anger advantage in spatially congruent conditions and a happiness advantage in spatially incongruent conditions. The funnel patterns in congruent and incongruent conditions are reversed in the second fixation, being compatible with a happiness advantage in spatially congruent conditions and an anger advantage in spatially incongruent conditions.

We corroborated these observations with a MAX*glmer* analysis on individuals’ fixation accuracy. This analysis included all factors in our experimental design, as well as the temporal order of the fixations encoded as a dichotomous covariate (Fixation Count = 1 for the First and 2 for the Second fixation) (*r*_c_ = 0.50, 95% CI [0.49, 0.52], see Table 2  Supplementary Materials). The results are consistent with an integrated SIA model with the values of the free parameters associated with PAF (*K*) and AAF (*c*) dependent on the fixation ordering according to a scanning of the visual scene from left-to-right. This was corroborated by a significant Target Position × Spatial Congruency × Fixation Count interaction, *F*(1, 4110) = 10.00, *p* = 0.001, η_p_^2^ = 0.312, 95% CI [0.037, 0.539]. This interaction was due to the different funnel pattern observed in the first and the second fixation. To better characterize such a difference, we ran separate analyses for the first and second fixation.

The MAX*glmer* model on first fixation accuracy (*r*_c_ = 0.52, 95% CI [0.49, 0.54], see Table 3 Supplementary Materials) revealed:

1. A general left-hemifield advantage, which was supported by a larger fixation accuracy for a target face presented on the left rather than on the right hemifield (mean for a target on the left = 0.63, mean for a target on the right = 0.46; difference = 0.17, 95% CI [0.13, 0.21], *t*(2100.60) = 7.86*, p* < 0.001; *d* = 0.34, 95% CI [0.26, 0.43]);
2. A funnel pattern in Congruent and Incongruent conditions, which was supported by a significant Intensity × Target Position × Spatial Congruency interaction, *F*(1, 44) = 15.00, *p* < 0.001, η_p_^2^ = 0.254, 95% CI [0.061, 0.437];
3. A spatial congruity anisotropy, which was supported by a significant Spatial Congruency × Target Position interaction (*F*(1, 2059) = 17.43, *p* < 0.001, η_p_^2^ = 0.284, 95% CI [0.080, 0.463].

The main effect of Average Emotion Intensity was not significant (*p* > *0.0*6), revealing the lack of the size effect.

The funnel pattern was characterized by a left-hemifield advantage (*c* = − 93.99 ± 42.58, *t*(32) = 2.21, *p* = 0.017; *d* = 0.38, 95% CI [0.03, 0.75]) combined with a happiness advantage (*K* = 41.38 ± 9.06, *t*(32) = 4.56, *p* < 0.001; *d* = 0.79, 95% CI [1.20, 0.40]). As consistent with the Spatial Congruency × Target Position interaction, the analysis of Michelson Contrast revealed an overall unbalance between* c* and *K*. This unbalance is representative of the unbalance between PAF and AAF. In particular, the unbalance was in favor of the AAF over PAF. This was revealed by a significant positive *M*_*c*_ (*M*_*c*_ = 0.41, 95% CI [0.23, 0.59], *t*(32) = 4.58, *p* < 0.001; *d* = 0.80, 95% CI [0.41, 1.20]).

The spatial congruity anisotropy is further supported by our post-hoc analyses that revealed a larger first fixations accuracy for emotional rather than intermediate targets, asymmetrically distributed over Spatial Congruency × Average Emotion Intensity conditions. In spatially congruent conditions, the anisotropy resulted in a funnel pattern compatible with an anger advantage (*k*_cong_ = − 52.60 ± 47.96, *t*(32) = − 1.10, *p* = *0.1*40; *d* = − 0.19, 95% CI [− 0.54, 0.16]). Whereas, in spatially incongruent conditions, the anisotropy resulted in a funnel pattern compatible with a happiness advantage (*k*_incong_ = 135.38 ± 38.60, *t*(32) = 3.51, *p* < 0.001; *d* = 0.61, 95% CI [0.24, 0.99]). The spatial congruity anisotropy was evidenced by the significant difference between *k*_cong_ and *k*_incong_ (difference = 187.99, 95% CI [64.88, 311.09], *t*(61.21) = 3.05,* p* = *0.0*03; *d* = 0.75, 95% CI [0.25, 1.25]). In spatially congruent conditions, fixations on emotional targets were more accurate than on intermediate targets, with a larger advantage observed in globally negative pairs (M_angriest|emotional_ = 0.60, M_happiest|intermediate_ = 0.45, difference = 0.16, 95% CI [0.07, 0.24], *t*(523.96) = 3.62, *p* < 0.001; *d* = 0.32, 95% CI [0.14, 0.49]) compared to globally positive pairs (M_angriest|intermediate_ = 0.59, M_happiest|emotional_ = 0.58; difference = 0.01, 95% CI [− 0.08, 0.09], *t*(522.00) = 0.18, *p* = *0.8*60; *d* = 0.02, 95% CI [− 0.16, 0.19]). Conversely, in spatially incongruent conditions, fixations on emotional targets were more accurate than on intermediate targets, with a larger advantage observed in globally positive pairs (M_angriest|intermediate_ = 0.39, M_happiest|emotional_ = 0.73; difference = − 0.34, 95% CI [− 0.26, − 0.42], *t*(520.61) = − 8.25, *p* < 0.001; *d* = − 0.72, 95% CI [− 0.54, − 0.89]), compared to globally negative pairs (M_angriest|emotional_ = 0.41, M_happiest|intermediate_ = 0.59; difference = − 0.17, 95% CI [− 0.09, − 0.26], *t*(524.99) = − 4.02, *p* < 0.001; *d* = − 0.35, 95% CI [− 0.18, − 0.52]).

The accuracy of the second fixation was analyzed following the exact same rationale as the one used for the first fixation (Fig. 2d–f). The MAX*glmer* model revealed an opposite pattern than the one observed on first fixation accuracy (*r*_c_ = 0.30, 95% CI [0.27, 0.32], see Table 4 Supplementary Materials). On the second fixation, participants were more accurate in detecting a target face displayed on the right rather than on the left hemifield (mean for a target on the left = 0.76, mean for a target on the right = 0.81; difference = − 0.06, 95% CI [− 0.09, − 0.02], *t*(2005.96) = − 3.11, *p* = 0.001; *d* = − 0.14, 95% CI [− 0.23, − 0.05]). We found:

1. A significant Average Emotion Intensity × Target Position × Spatial Congruency interaction (*F*(1, 44) = 16.42, *p* < 0.001, η_p_^2^ = 0.272, 95% CI [0.072, 0.453]), which was consistent with a funnel pattern.
2. A main effect of Average Emotion Intensity (*F*(1, 1984) = 63.62, *p* < 0.001, η_p_^2^ = 0.591, 95% CI [0.386, 0.706]), which was supported by a size effect in which fixation accuracy increased as Average Emotion Intensity grew larger, *β* = 0.37 ± 0.15, *t*(33.47) = 2.36, *p* = 0.018, from negative to positive Average Emotion Intensity.

The results did not show any other significant main effects or interactions (*p* > 0.06).

The funnel pattern was characterized by a right-hemifield advantage (*c* = 24.43 ± 18.68, *t*(32) = 1.31, *p* = *0.1*00; *d* = 0.23, 95% CI [− 0.12, 0.58]), and a null happiness advantage (*K* = 1.47 ± 18.68, *t*(32) = 0.11, *p* = 0.456; *d* = 0.02, 95% CI [− 0.33, 0.37]). Again, the PAF and AAF resulted to be unbalanced in favor of AAF (*M*_*c*_ = 0.22, 95% CI [0.10, 0.35], *t*(32) = 3.64, *p* < 0.001; *d* = 0.63, 95% CI [0.26, 1.02]).

We corroborated the spatial congruity anisotropy by post-hoc analyses. In spatially congruent conditions, we found a funnel pattern compatible with a happiness advantage (*k*_cong_ = 25.90 ± 30.78, *t*(32) = 0.84, *p* = *0.2*03; *d* = 0.15, 95% CI [− 0.20, 0.50]). Conversely, in spatially incongruent conditions, the funnel pattern was compatible with an anger advantage (*k*_incong_ = − 22.95 ± 9.84, *t*(32) = − 2.33, *p* = 0.013; *d* = − 0.41, 95% CI [− 0.77, − 0.05]). The difference between *k*_cong_ and *k*_incong_ (difference = 48.85, 95% CI [131.55, 7.32], *t*(36.84) = 2.27, *p* = 0.029; *d* = 0.57, 95% CI [− 1.07, − 0.06]) was significant. In spatially congruent conditions, fixation accuracy for emotional rather than intermediate targets was larger in globally positive pairs (M_angriest|intermediate_ = 0.80, M_happiest|emotional_ = 0.88; difference = − 0.08, 95% CI [− 0.15, − 0.02], *t*(478.26) = − 2.57, *p* = 0.011; *d* = − 0.23, 95% CI [− 0.41, − 0.05]) compared to globally negative pairs (M_angriest|emotional_ = 0.71, M_happiest|intermediate_ = 0.70; difference = 0.02, 95% CI [− 0.06, 0.09], *t*(512.99) = 0.40, *p* = *0.6*91; *d* = 0.04, 95% CI [− 0.14, 0.21]). Conversely, for spatially incongruent conditions, fixation accuracy for emotional rather than intermediate targets was larger in globally negative pairs (M_angriest|emotional_ = 0.82, M_happiest|intermediate_ = 0.63; difference = 0.19, 95% CI [0.26, 0.11], *t*(480.17) = 4.88, *p* < 0.001; *d* = 0.43, 95% CI [0.61, 0.26]) compared to globally positive pairs (M_angriest|intermediate_ = 0.88, M_happiest|emotional_ = 0.85; difference = 0.03, 95% CI [− 0.03, 0.09], *t*(503.16) = 0.92, *p* = *0.3*58; *d* = 0.08, 95% CI [− 0.09, 0.26]).

The final analysis aimed to test the accuracy of integrated SIA predictions on each fixation. This was done by remapping the individual values associated with the experimental factors (such as emotional intensity, spatial congruency, and target position) separately for the first and second fixation. The most parsimonious linear combination of SIA intensity components was applied to each individual's pattern of accuracy. More information on this analysis can be found in the Data analysis subsection of the manuscript.. SIA remapped values accounted for both the pattern of accuracy of the first (with empirically determined parameters* K* = 41.38,* c* = − 93.99 e α = 0.4) and the second (with empirically determined parameters* K* = 1.47,* c* = 24.00 e α = 1.2) fixation. The remapped values were the only significant factor of the MAX*glmer* in both the First (*F*(1, 2085) = 32.07*, p* < *0.0*01, η_p_^2^ = 0.640, 95% CI [0.301, 0.775]) and Second (*F*(1, 2010) = 76.86, *p* < *0.0*01, η_p_^2^ = 0.810, 95% CI [0.581, 0.881]) fixation. The remapped values nullified the Average Emotion Intensity × Target Position × Spatial Congruency interaction in both fixation analyses.

## Discussion

We reported an experiment that examined the link between pairs of emotional faces shown side-by-side, and the eye-movement dynamics involved in gazing as a probe of choice behavior. We proposed and tested whether the prioritization of attention from the perceptual salience of facial expressions of emotion (PAF) and the dependence of attention from the scanning habit (AAF, prioritizing faces lying on the left or right visual hemifield depending on the gazing temporal phase) could account for comparative judgments of emotions selected by gazing. In particular, the results show that low-level attentional factors alone can account for gaze-contingent comparative choice in emotional pairs, independent of high-level knowledge-based factors that are typically invoked to account for results in manual simultaneous comparison tasks^8–13,29,49,50,61^.

Inspired by Schurgin et al.^48^ (but see also^52–54^), we analyzed the informative part of gazing behavior. We analyzed the pattern of fixation accuracy and dwell time separately, by first pooling the first two fixations and then analyzing them. Fixation accuracy, as well as dwell time and saccade latencies, produce a pattern of results which is similar to the one observed by Fantoni et al.^18^ in bimanual simultaneous comparison tasks of facial expressions of emotion. Fixation accuracy and dwell times, similar to the speed of manual choice between the happiest or the angriest face within a pair, were found to be larger for facial expressions displaying the most intense emotion within the pair. Specifically, we observed that fixation accuracy and dwell times increased as the absolute emotion intensity of the selected face increased, as well as the average emotion intensity of the pair. This corresponded to a funnel pattern in the domain of gazing.

We interpreted this pattern as consistent with PAF, the perceptual salience of emotional stimuli causes emotional faces to capture more attention than neutral faces, regardless of their emotional valence (being it negative as with angry faces or positive as with happy faces). This is consistent with a 3-way interaction between Average Emotion Intensity, Target Position and Spatial Congruency, with the lines connecting fixation accuracy and dwell time for the Left and Right targets crossing over when plotted against Average Emotion Intensity. The cross point of such a funnel pattern was compatible with a facilitation for the positive over negative emotions (i.e. a happiness advantage) in spatially incongruent conditions and a facilitation for the negative over positive emotions (i.e. an anger advantage) in spatially congruent conditions. We named this difference between congruent and incongruent conditions as the spatial congruity anisotropy. This anisotropy is consistent with a modulation of PAF by AAF. AAF produces a left hemifield advantage with a larger accuracy of fixation for targets displayed to the left rather than the right.

The analysis of the pattern of gaze-contingent choice over time (first and second fixation) revealed a similarity between the specific pattern of spatial congruity anisotropy found on the first fixation and that on the second fixation. However, this similarity was reversed, with an anger advantage in spatial incongruent conditions and a happiness advantage in spatial congruent conditions on the first fixation and a happiness advantage in spatial incongruent conditions and an anger advantage in spatial congruent conditions on the second fixation.

We hypothesized and found that the different patterns of spatial congruity anisotropy, found in the present experiment as well as in Fantoni et al.^18^, are accounted for by an integrated SIA model. The integrated SIA model formalizes a general interpretative framework for the origin of the funnel pattern in emotion comparison tasks based on purely low-level factors like PAF and AAF. The integrated SIA model includes AAF driven by the scanning habit as an additional free parameter to PAF that was already modelled by the standard SIA^18,19^. In particular, the pattern of data at the beginning of the informative part of gazing behavior (i.e. the first fixation) is consistent with an integrated SIA model, which produces an overall facilitation for targets displayed on the left visual hemifield rather than the right. Additionally, this pattern is also consistent when a display is tachistoscopically presented^18^. This effect combined with the crossover patter predicted by PAF alone results in a funnel pattern compatible with an anger advantage in spatially congruent conditions and a happiness advantage in spatially incongruent conditions. In contrast, the fixation accuracy pattern observed at the end of the informative part of gazing behavior, namely the second fixation, can be explained by an integrated SIA model, which results in a hindering effect for targets displayed on the left visual hemifield compared to the right hemifield. This is consistent with a funnel pattern compatible with a happiness advantage in spatially congruent conditions *vs.* an anger advantage in spatially incongruent conditions. The integrated SIA model fully accounts for both the patterns of fixation accuracy over time (first and second fixation separately) and for their pooled pattern. This was shown by the high predictive power of the remapping of emotional pairs in terms of the simple sum between Target Absolute Emotion Intensity + Average Emotion Intensity × size effect + Emotion anisotropy + Left-to-Right scanning habit.

The optimal values of AAF to account for our pattern of data over the first and second fixation are consistent with a scanning habit developing over time from left to right. This scanning habit likely corresponded to the reading direction of our Italian sample of participants. Given such correspondence, the results of our study cannot be considered as determinant to establish whether the direction of scanning of the visual scene in our task is driven by cultural or innate factors. Further research is needed to answer such an issue possibly investigating our attentional bias on a sample of participants from cultures where the dominant writing system is from right-to-left (e.g. Arabic, Hebrew).

How can we reconcile our finding showing that the choice in emotion comparison is purely driven by low-level components of attention beyond high-level components?

The discovery of the semantic congruity effect^8^ has sparked a theoretical debate regarding the nature of the comparison process that produces the observed choice pattern in comparison tasks. Specifically, the debate focuses on whether the process it is driven by high-level or low-level cognitive processes. The standard interpretation of the pattern of choice observed in comparison tasks has been conceived by many researchers^8,12–16^ as the by-product of the congruity between different types of properties of the stimulus (e.g. quantity, lightness, spatial position, size, emotion, etc.) and their high-level semantic representations (i.e. the one defined by the task or the implicit magnitude representation of the corresponding stimulus properties). The standard approach to interpret these congruity effects assumes an explicit scheme of knowledge about the association between stimulus properties and its implicit magnitude representations. Remarkable examples of this approach have been applied to the representation of numbers (e.g. Ref.^12^), spatial position (e.g. Ref.^18^), size (e.g. picture/words of animals^13^), age (e.g. Ref.^62^), probabilities of events (e.g. Ref.^63^), skin color^61^, sound level pressure of acoustic stimuli^64^, temperature^16^, brightness^17,65^, height, depth, size, and width^66^.

However, our results, together with the general framework operationalized by the integrated SIA model, provide a general way to interpret the results of comparison tasks, regardless of high-level semantic knowledge. This is based on cognitive mechanisms involving low-level attentional factors alone. Such mechanisms require a basic cognitive system at work. This idea is consistent with recent research in which the same pattern of results is found in pre-linguistic toddlers^23,65^ and animals like monkeys^14,67^. SIA can be used to interpret the pattern of choice in comparison tasks regardless of the type of instruction being used, and any representation of stimulus intensity along a unidimensional magnitude continuum. The way AAF and PAF are modelled by SIA suggests that the choice of emotion, even in an abstract situation as the one characterized by the simultaneous comparison of two facial expressions, cannot be labelled univocally in favor of one specific emotional valence. This applies to neither positive emotions, known to produce a happiness advantage, nor to negative emotions, known to produce an anger advantage. Our results indeed demonstrate that the advantage of one emotional valence over the other depends on stimulus-driven attentional factors that are likely to depend on the perceptual salience of emotions (as extracted from perceptual cues associated with facial expression) and the temporal dynamic of gazing behavior (as prioritizing one hemifield over the other in different temporal phases). In particular, the specific temporal phase of the gazing (whether at the beginning or at the end of the exploration) can turn a happiness advantage into an anger advantage (and vice-versa), making the labelling of the happiness or the anger advantage valuable only on relativistic (not absolute) terms. Future studies could investigate the generalizability of the AAF driven by scanning habits, as well as extend our study of emotion comparison tasks to test how fixation and dwell time patterns may be affected by other spatial orientations of the emotional pairs than the horizontal one. For example, studies could explore how the vertical orientation of faces arranged one above the other might impact the observed patterns.

To conclude, our results show that the role played by high-level semantic and linguistic cognitive factors in patterns of data generally interpreted as the result of comparative judgements can be accounted for by the incidental combination of purely stimulus-driven, exogenous *perceptual* (PAF) and purely stimulus-independent, endogenous *automatic* attentional factors (AAF). These two factors may constitute the evolutionary bricks of the well-known semantic congruity effect. The way these two factors may be operationally combined in order to be predictive about different aspects of the choice in comparative judgments (i.e., the speed, the accuracy as well as the latency of choice) as modelled by the SIA, may be used to instruct object classification algorithms to make their discrimination capabilities more efficient and faster in situations involving the simultaneous comparison of multiple options. This could be beneficial in situations where fast and accurate decision-making is necessary, such as in medical diagnosis or security screening.

## Supplementary Information

Supplementary Information 1.

Supplementary Information 2.

## Data Availability

All raw data generated in this study are included in this published article as supplemental materials file (Raw_Data.xlsx).
