# Supplementary material for: The temporal dynamics of emotion comparison depends on low-level attentional factors
Source: Sci Rep. 2023 May 5;13:7324. doi: 10.1038/s41598-023-33711-0 (PMC10163009; doi:10.1038/s41598-023-33711-0)
Supplement: Supplementary file 2 — Supplementary Information 2. [file 41598_2023_33711_MOESM2_ESM.docx]

Supplementary Materials to
“The temporal dynamics of emotion comparison depends on low-level attentional factors”

Andrea Dissegna^1^, Giulio Baldassi^1^, Mauro Murgia^1^, Francesco Darek Costa^1^, & Carlo Fantoni^1^

^1^ Department of Life Sciences, University of Trieste

Authors note

Andrea Dissegna: <https://orcid.org/0000-0002-4333-3353>

Giulio Baldassi: <https://orcid.org/0000-0002-2114-3567>

Mauro Murgia: <https://orcid.org/0000-0002-8450-1157>

Francesco Darek Costa: https://orcid.org/0000-0002-8299-5819

Carlo Fantoni: <https://orcid.org/0000-0002-7749-1771>

Correspondence concerning this article should be addressed to Carlo Fantoni, Via E. Weiss 21, 34128 Trieste, Italy. E-mail: [cfantoni@units.it](mailto:cfantoni@units.it)

# **Analysis of Fixation accuracy**

## Descriptive statistics

Participants’ average number of fixations was 3.15 (min-max range = 1-11, SD = 1.91). Their average fixation accuracy rate was 81% (SD = 0.39). The accuracy increased steadily from the first fixation (mean accuracy = 54%, SD = 0.50) to the second fixation (mean accuracy = 78%, SD = 0.41).

## MAXglmer models

**Table 1SM.** The summary table of the MAXglmer model including Average Emotion Intensity × Target Position × Spatial Congruency. The random structure was: Average Emotion Intensity × Target Position × Spatial Congruency | Subj.

|  | **Fixation accuracy** | | |
| --- | --- | --- | --- |
| *Predictors* | *Estimates* | *CI* | *p* |
| (Intercept) | 0.44 | 0.25 – 0.64 | **<0.001** |
| Target Position [RIGHT] | -0.25 | -0.51 – 0.01 | 0.061 |
| Congruency [INCONG] | -0.15 | -0.33 – 0.02 | 0.088 |
| Average emotion intensity [+50] | 0.09 | -0.08 – 0.26 | 0.292 |
| Target Position [RIGHT] × Congruency [INCONG] | 0.27 | 0.04 – 0.50 | **0.020** |
| Target Position [RIGHT] × Average emotion intensity [+50] | 0.40 | 0.15 – 0.65 | **0.002** |
| Congruency [INCONG] × Average emotion intensity [+50] | 0.58 | 0.32 – 0.85 | **<0.001** |
| Target Position [RIGHT] × Congruency [INCONG]) × Average emotion intensity [+50] | -1.06 | -1.43 – -0.69 | **<0.001** |

**Table 2SM.** The summary table of the MAXglmer model including Average Emotion Intensity × Target Position × Spatial Congruency × Fixation count. The random structure was: Average Emotion Intensity × Target Position × Spatial Congruency × Fixation Count | Subj.

|  | **Fixation accuracy** | | |
| --- | --- | --- | --- |
| *Predictors* | *Estimates* | *CI* | *p* |
| (Intercept) | 0.27 | 0.10 – 0.44 | **0.002** |
| Target position [RIGHT] | -0.40 | -0.62 – -0.19 | **<0.001** |
| Congruency [INCONG] | -0.05 | -0.27 – 0.17 | 0.665 |
| Average emotion intensity [50] | -0.02 | -0.28 – 0.24 | 0.866 |
| Fixation count [2] | 0.32 | 0.10 – 0.55 | **0.005** |
| Target position [RIGHT] × Congruency [INCONG] | -0.04 | -0.35 – 0.27 | 0.802 |
| Target position [RIGHT] × Average valF [50] | 0.39 | 0.03 – 0.76 | **0.035** |
| Congruency [INCONG] × Average emotion intensity [50] | 0.50 | 0.13 – 0.86 | **0.009** |
| Target position [RIGHT] × Fixation count [2] | 0.35 | 0.03 – 0.67 | **0.032** |
| Congruency [INCONG] × Fixation count [2] | -0.19 | -0.51 – 0.13 | 0.238 |
| Average emotion intensity [50] × Fixation count [2] | 0.35 | 0.01 – 0.68 | **0.041** |
| Target position [RIGHT] × Congruency [INCONG]) × Average emotion intensity [50] | -0.94 | -1.46 – -0.42 | **<0.001** |
| Target position [RIGHT] × Congruency [INCONG]) × Fixation count [2] | 0.68 | 0.23 – 1.14 | **0.003** |
| Target position [RIGHT] × Average emotion intensity [50]) × Fixation count [2] | 0.08 | -0.41 – 0.56 | 0.754 |
| Congruency [INCONG] × Average emotion intensity [50]) × Fixation count [2] | 0.14 | -0.35 – 0.62 | 0.574 |
| Target position [RIGHT] × Congruency [INCONG] × Average emotion intensity [50]) × Fixation count [2] | -0.30 | -1.00 – 0.39 | 0.389 |

**Table 3SM.** The summary table of the MAXglmer model performed on the first fixation and including Average Emotion Intensity × Target Position × Spatial Congruency. The random structure was: Average Emotion Intensity × Target Position × Spatial Congruency | Subj.

|  | **Fixation accuracy** | | |
| --- | --- | --- | --- |
| *Predictors* | *Estimates* | *CI* | *p* |
| (Intercept) | 0.32 | 0.31 – 0.32 | **<0.001** |
| Target position [RIGHT] | -0.58 | -0.59 – -0.58 | **<0.001** |
| Congruency [INCONG] | -0.03 | -0.03 – -0.03 | **<0.001** |
| Average emotion intensity [50] | -0.03 | -0.04 – -0.03 | **<0.001** |
| Target position [RIGHT] × Congruency [INCONG] | 0.02 | 0.02 – 0.02 | **<0.001** |
| Target position [RIGHT] × Average valF [50] | 0.53 | 0.53 – 0.53 | **<0.001** |
| Congruency [INCONG] × Average emotion intensity [50] | 0.62 | 0.62 – 0.62 | **<0.001** |
| (Target position [RIGHT] × Congruency [INCONG]) × Average emotion intensity [50] | -1.27 | -1.27 – -1.26 | **<0.001** |

**Table 4SM.** The summary table of the MAXglmer model performed on the second fixation and including Average Emotion Intensity × Target Position × Spatial Congruency. The random structure was: Average Emotion Intensity × Target Position × Spatial Congruency | Subj.

|  | **Fixation accuracy** | | |
| --- | --- | --- | --- |
| *Predictors* | *Estimates* | *CI* | *p* |
| (Intercept) | 0.67 | 0.38 – 0.96 | **<0.001** |
| Target position [RIGHT] | -0.08 | -0.48 – 0.32 | 0.690 |
| Congruency [INCONG] | -0.26 | -0.54 – 0.01 | 0.057 |
| Average emotion intensity [50] | 0.38 | 0.06 – 0.69 | **0.018** |
| Target position [RIGHT] × Congruency [INCONG] | 0.67 | 0.27 – 1.07 | **0.001** |
| Target position [RIGHT] × Average emotion intensity [50] | 0.35 | -0.10 – 0.80 | 0.123 |
| Congruency [INCONG] × Average emotion intensity [50] | 0.56 | 0.10 – 1.02 | **0.017** |
| Target position [RIGHT] × Congruency [INCONG]) × Average emotion intensity [50] | -1.15 | -1.78 – -0.52 | **<0.001** |

# **Analysis of Dwell times**

## Descriptive statistics

Participants dwell on faces for an average time of 1443.8 ms (min-max range = 21-6011, SD = 1271.06). The dwell time on the first fixation was 1201.59 (SD = 1315.39), and it increased to 1772.07 (SD = 1236.50) on the second fixation.

## Overall dwell times

Figure 1SM depicts the participants’ dwell times aggregated over the first two fixations. The pattern of data closely resembles the standard pattern of motor reactivity and accuracy observed by Fantoni et al. (2019) and is similar to the one resulting from the analysis of fixation accuracy. The pattern of dwell times is characterized by a funnel pattern, with the best *lmer* regression lines connecting dwell times for left (grey lines) and right (yellow lines) targets crossing over in a true interaction when plotted against average emotion intensity in both congruent (panel a) and incongruent (panel b) spatial positions. The overall pattern of dwell time revealed a rather symmetric funnel pattern, which is consistent with a happiness advantage in both spatially congruent and incongruent conditions. The happiness advantage was indicated by the *lme* regressors crossing at negative values of average emotion intensity. The funnel pattern observed in congruent and incongruent conditions is fully predicted by the values of individuals’ fixation accuracy, which were remapped according to the most parsimonious linear combination of intensity components predicted by an integrated SIA, given the individual values of dwell time (Figure 1SM, panel b: empirically determined free parameters: *K* = 7.74, *c* = 3.28, and α = 0.6).


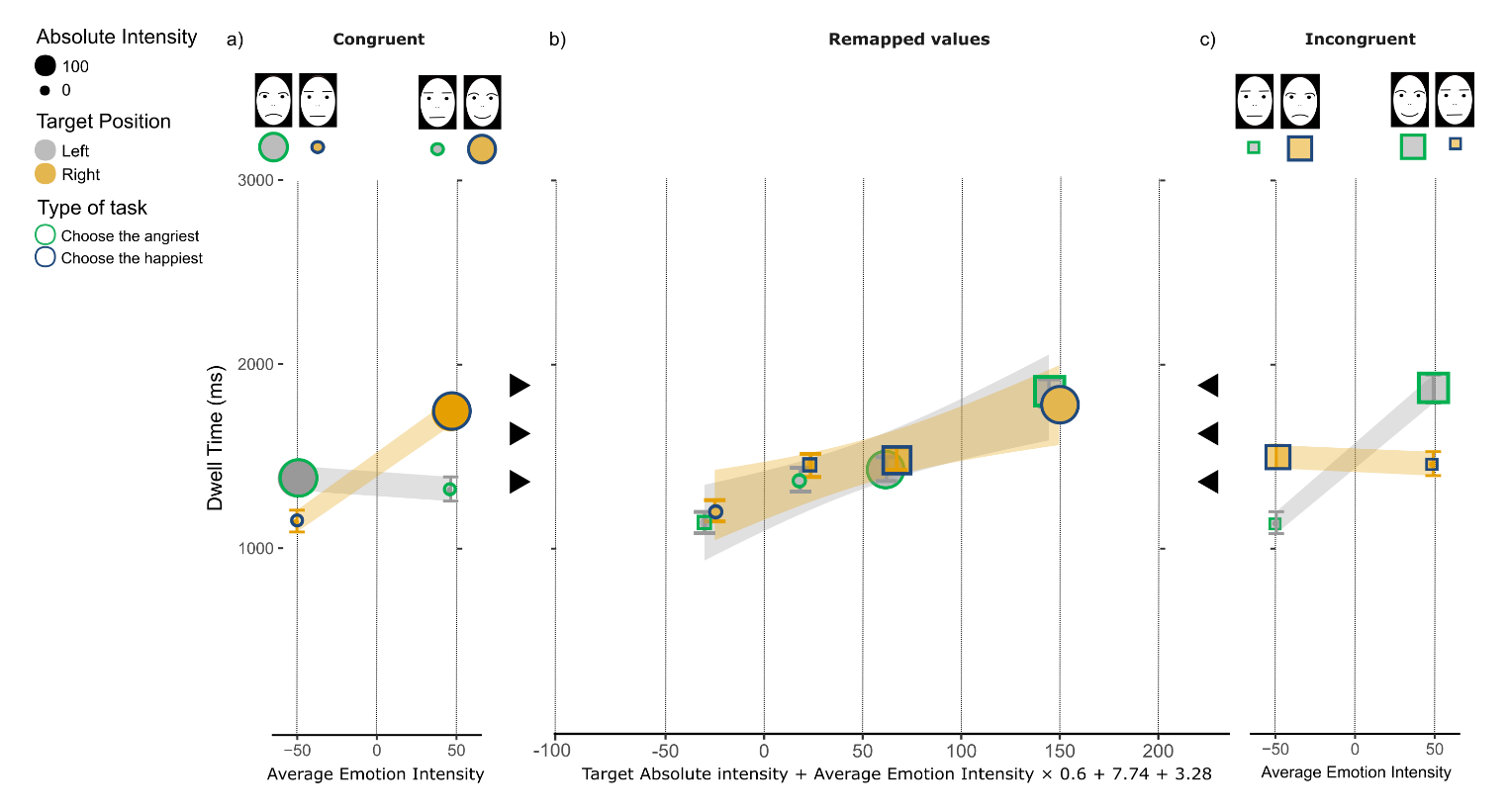
**Fig 1SM.** Pooled distribution of dwell times across the first two fixations. (a-c), illustration of the mean dwell time in Spatially Congruent (circles in panel a) and Spatially Incongruent (squares in panel c) conditions. Within each panel, mean dwell times are shown as a function of Average Emotion Intensity (panels a and c) and as a function of the best SIA remapped values (panel b). SIA remapped values of experimental condition intensities were obtained including the 3 empirically determined values modeling: (1) the size effect, (2) the emotion anisotropy and the (3) the scanning habit (equation below the *x-axis* from the leftmost to the rightmost free parameter). Error bars represent ± 1 s.e.m. and the size of the circles represent the absolute emotion intensity (small = intermediate; large = 100 per cent angry or 100 per cent happy). The Target Position and the type of task are coded by the colors filling and bounding the circles, respectively (legend). Gray/yellow lines in panels a-c are the best fitting MAX*lmer* regression lines for Left/Right Target Position conditions, with the shaded bands corresponding to ± 1 standard error of the regression. On the top of panels a and c, the schematic representations of the photographs of human facial expressions that we used in the experiment (for more details see the Material subsection of the manuscript).

These observations were corroborated by a MAX*lmer* model (*r*_c_ = .24, 95% CI [.22, .26], see Table 5SM in the MAX*lmer* models section). The model revealed:

1. an Average Emotion Intensity × Target Position × Spatial Congruency interaction, *F*(1, 74.77) = 60.54, *p* < .001, η_p_^2^ = .45, 95% CI [.28, .58]. This interaction was consistent with a funnel pattern in both congruent and incongruent spatial positions.
2. A main effect of the factor Average Emotion Intensity (*F*(1, 64.22) = 45.74, *p* < .001, η_p_^2^ = .42, 95% CI [.24, .56]). This was consistent with a size effect.. Participants dwell time increased from globally negative to globally positive face pairs: *β* = 53.01 ± 94.571, *t*(3123.4) = 6.21, *p* < 0.001, *d =* 0.22, 95% CI [0.15, 0.29].

No other main effect or interaction was significant (*p >* .06), including the Spatial Congruency × Target Position interaction (*F*(1, 30.81) = 1.10, *p* = .301), diagnostic of an emotion anisotropy.

The funnel pattern was characterized by a null right or left hemifield advantage (*c* = 3.28 ± 6.20, *t*(30) = 0.48, *p* = .318; *d* = 0.09, 95% CI [-0.27, 0.45]) and a happiness advantage (*K* =7.74 ± 7.46, *t*(30) = 1.32, *p* = .098; *d* = 0.24, 95% CI [-0.12, 0.60]). Overall, AAF and PAF were balanced, as consistent with a null M_c_ (*M_c_* = -.09, 95% CI [-0.07, 0.26], *t*(30) = 0.87, *p* = .389; *d* = 0.16, 95% CI [-0.20, 0.52]). The null M_c_ indicates a symmetric funnel pattern across spatial congruent and incongruent conditions. The lack of spatial congruity anisotropy was further corroborated by the analysis on the crosspoint between best fitting *lmer* in congruent *k*_cong_ and incongruent spatial position *k*_incong_. In both spatially congruent and incongruent conditions, the funnel was compatible with a happiness advantage (*k*_cong_ = 11.03±10.68, *t*(30) = 1.09, *p* = .141; *d* = 0.20, 95% CI [-0.16, 0.56]; *k*_incong_ = 4.46 ± 8.86, *t*(30) = 0.52, *p* = .696; *d* = 0.09, 95% CI [-0.26, 0.45]). The difference between *k*_cong_ and *k*_incong_ was not significant (difference = -6.57, 95% CI [-34.06, 20.92], *t*(57.43) = -0.48, *p* = .634; *d* = -0.12, 95% CI [-0.62, 0.38]). This further corroborates the lack of difference in the funnel pattern observed in both Spatial Congruency conditions.

Additional evidence of the lack of spatial congruity anisotropy resulted from the post-hoc analyses of dwell times. In spatially congruent conditions, dwell times on emotional targets were larger than on intermediate targets, in both negative (M_angriest|emotional_ = 1418.67 ± 66.80, vs. M_happiest|intermediate_ = 1186.02 ± 59.49, *χ^2^*(1) = 5.31, *p* = 0.02, *d* = 0.89) and positive Average Emotion Intensity pairs (M_angriest|emotional_ = 1360.14 ± 65.78, vs. M_happiest|intermediate_ = 1777.18 ± 73.13, *χ^2^*(1) = 16.37, *p* < .001, *d* = 2.04). We obtained similar results in spatially incongruent conditions. Dwell times on emotional targets were larger than on intermediate targets in both negative (M_angriest|emotional_ = 1477.37 ± 62.78, vs. M_happiest|intermediate_ = 1119.38 ± 59.52, *χ^2^*(1) = 10.83, *p* < .001, *d* = 1.43) and a positive Average Emotion Intensity pairs (M_angriest|emotional_ = 1439.63 ± 64.87, vs. M_happiest|intermediate_ = 1844.81 ± 77.13, *χ^2^*(1) = 14.52, *p* < .001, *d* = 1.82).

As a final analysis, we quantitatively tested the goodness of the integrated SIA predictions (details in the main text, Data analysis subsection). The integrated SIA remapped values with empirically determined parameters *K* = 7.74, *c* = 3.28 and α = 0.6, were the only significant factor (*F*(1, 79.54) = 10.39, *p* = .001, η_p_^2^ = 0.12, 95% CI [.02, .26]) of the MAX*lmer* model, nulling the Target Position × Spatial Congruency × Average Emotion Intensity interaction (*F*(1, 70.63) = 3.87, *p* = .825).

## Dwell times over time

Figure 2SM shows the distribution of the first (panels a-c) and second (panels d-f) fixations’ dwell time as a function of average emotion intensity for spatially congruent (panel a-d) and incongruent pairs (panel c-f). The same distribution of dwell time was plotted as a function of integrated SIA remapped values in panels b) and e). Notably, the funnel patterns observed on the first fixation (Figure 2SM, panels a-c) are different from those observed on the second fixation (Figure 2SM, panels d-f). The distribution of dwell times on the First fixations is characterized by a funnel pattern compatible with an anger advantage in spatially congruent conditions and a happiness advantage in spatially incongruent conditions. The funnel patterns in congruent and incongruent conditions are reversed on the second fixation, being compatible with a a happiness advantage in spatially congruent conditions and an anger advantage in spatially incongruent conditions.

**
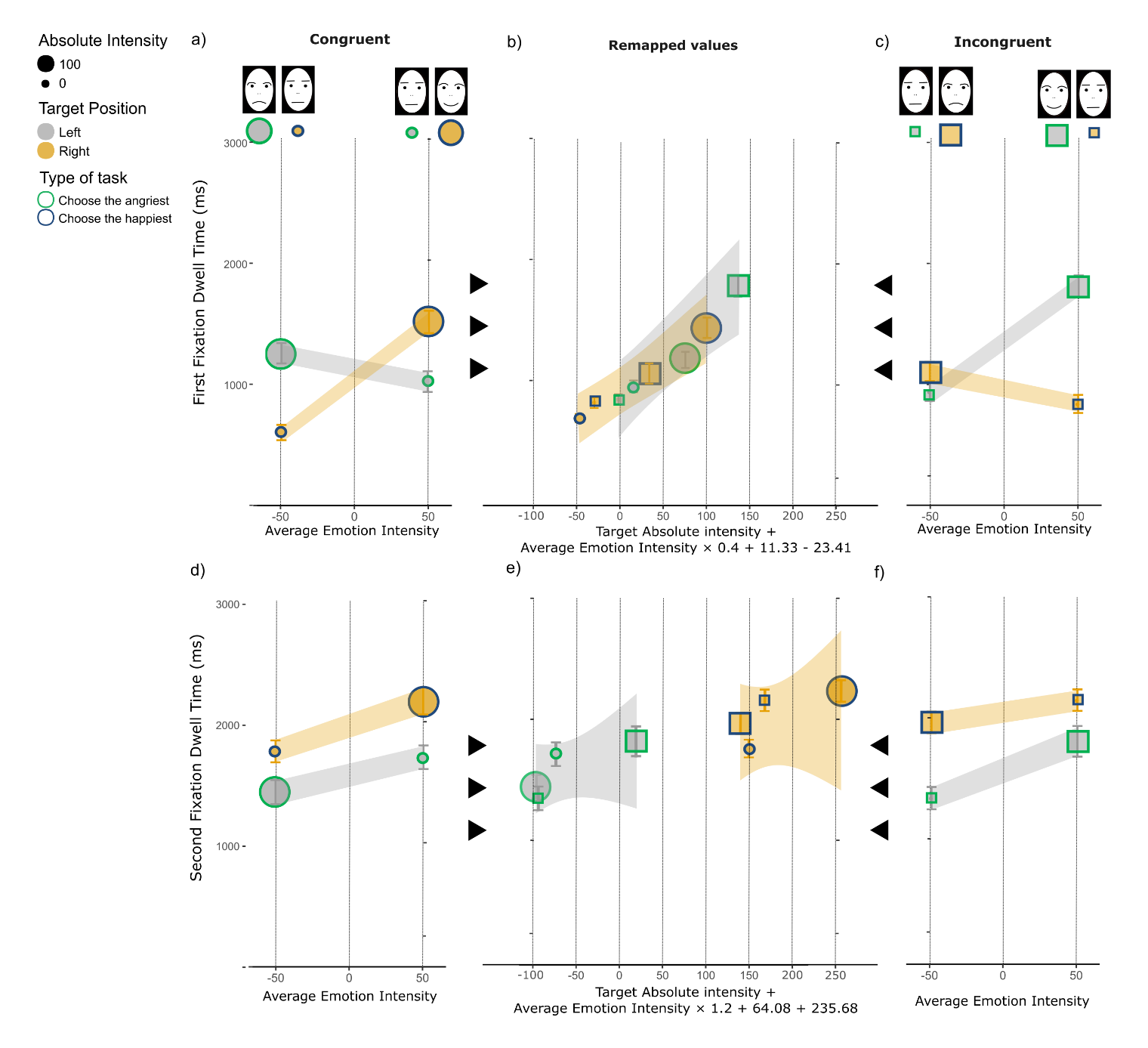
Fig 2SM.** Distribution of First and Second fixations’ dwell times. (a-c) depiction of the mean of the first and second fixations’ dwell times (d-f) in Spatially Congruent (panel a and d) and Spatially Incongruent (panel c and f) conditions. Within each panel, mean dwell times are shown as a function of Average Emotion Intensity (panels a, c, d and f) and as a function of the best SIA remapped values (panels b and e). SIA remapped values of experimental conditions intensities were obtained including the 3 empirically determined values modeling (1) the size effect, (2) the emotion anisotropy and the (3) the scanning habit (equation below the *x-axes* from the leftmost to the rightmost free parameter).. The same symbols and color encoding of variables used in Figure 1SM is used as by the legend on top with error bars indicating ± 1 standard error of the mean and gray/yellow lines in panels a-f are the best fitting MAX*lmer* regression lines for Left/Right Target Position conditions, with the shaded bands corresponding to ± 1 standard error of the regression. On the top of panels a and c, the schematic representations of the photographs of human facial expressions that we used in the experiment (for more details see the Material subsection of the manuscript).

Similar to the results of the analysis on fixation accuracy, the MAX*lmer* model, which included Average Emotion Intensity, Target Position, Spatial Congruency as fixed effects, and the temporal order of fixations (Fixation Count) as a dichotomous covariate (*r*_c_ = .49, 95% CI [.47, .51], see Table 6SM in the MAXlmer models section) revealed a significant Average Emotion Intensity × Target Position × Spatial Congruency × Fixation Count interaction, *F*(1, 46.29) = 26.78, *p* < .001, η_p_^2^ = .37, 95% CI [.16, .54]. This interaction was due to the different funnel patterns observed in the first and the second fixation. To better characterize such difference, we ran separate analysis for the first and second fixation. In both cases, we modelled the pattern of dwell times using a MAX*lmer* model with the same structure of the one used for the aggregated data.

The MAX*lmer* model (*r*_c_ = .46, 95% CI [.43, .49], see Table 7SM in the MAX*lmer* models section) revealed:

1. a general left-hemifield advantage, which was consistent with longer dwell times for a target face presented on the left rather than on the right hemifield (M_left_ = 1589.09, M_right_ = 1296.28, difference = 292.81, 95% CI [200.46, 385.15], *t*(3123.37) = 6.22, *p* < .001; *d* = 0.22, 95% CI [0.15, 0.29]);
2. a significant Average Emotion Intensity × Target Position × Spatial Congruency interaction, *F*(1, 57.69) = 95.67, *p* < .001, η_p_^2^ = .62, 95% CI [.47, .73], which was consistent with a funnel pattern in Congruent and Incongruent conditions;
3. a main effect of Average Emotion Intensity, *F*(1, 29.75) = 30.52, *p* < .001, η_p_^2^ = .511, 95% CI [.241, .684], which was consistent with a size effect in the domain of emotions. Participants dwell times increased from globally negative to globally positive face pairs with a rate of *β* = 227.10± 0.086, *t*(66.54) = 2.06, *p* = 0.043.

No other main effect or interaction was significant (*p >* .06), including the Spatial Congruency × Target Side interaction (*F*(1, 4088) = -2.67, *p* = .08), diagnostic of an emotion anisotropy.

The funnel pattern was characterized by an overall left-hemifield advantage (*c* = -23.41±9.61, *t*(30) = -2.38, *p* = .012; *d* = -0.43, 95% CI [-0.80, -0.06]) combined with a happiness advantage (*K* = 11.33±5.51, *t*(30) = 2.16, *p* = 0.019; *d* = 0.39, 95% CI [0.02, 0.76]). The analysis of the Michelson Contrast revealed an overall unbalance between AAF and a PAF, in favor of AAF (*M_c_* = 0.22, 95% CI [0.09, 0.43], *t*(32) = 2.13, *p* = .041; *d* = 0.38, 95% CI [0.02, 0.76]).

The presence of a spatial congruity anisotropy is further supported by post-hoc analyses. The post-hoc analysis of the first fixation revealed larger dwell times for emotional targets compared to intermediate targets, asymmetrically distributed over Spatial Congruency × Average Emotion Intensity conditions. In spatially congruent conditions, the anisotropy resulted in a funnel pattern compatible with an anger advantage (*k*_cong_ = -12.05 ± 10.49, *t*(30) = -1.08, *p* = 0.145; *d* = -0.19, 95% CI [-0.56, 0.17]). Conversely, in spatially incongruent conditions, the funnel pattern was compatible with a happiness advantage (*k*_incong_ = 34.76 ± 11.65, *t*(30) = 2.98, *p* = .003; *d* = 0.54, 95% CI [0.16, 0.92]). The spatial congruity anisotropy was evidenced by the significant difference between the *k*_cong_ vs *k*_incong_ (difference = 46.82, 95% CI [15.45, 78.18], *t*(59.35) = 2.99, *p* = 0.004; *d* = 0.76, 95% CI [0.24, 1.27]). In spatially congruent conditions, the advantage for emotional rather than intermediate targets was larger in globally negative pairs (M_angriest|emotional_ = 1390.14, M_happiest|intermediate_ = 700.00; difference = 690.14, 95% CI [478.66, 901.62], *t*(443.65) = 6.41, *p* < .001; *d* = 0.58, 95% CI [0.40, 0.76]) compared to globally positive pairs (M_angriest|intermediate_ = 1157.66, M_happiest|emotional_ = 1601.93; difference = -444.27, 95% CI [-693.85, -194.69], *t*(482.91) = -3.50, *p* < .001; *d* = -0.32, 95% CI [-0.49, -0.14]). In spatially incongruent conditions, the advantage for emotional rather than intermediate targets was larger in globally positive pairs (M_angriest|intermediate_ = 922.40, M_happiest|emotional_ = 1861.98; difference = -939.58, 95% CI [-700.55, -1178.61], *t*(470.99) = -7.72, *p* < .001; *d* = -0.70, 95% CI [-0.51, -0.88]) compared to globally negative pairs (M_angriest|emotional_ = 969.10, M_happiest|intermediate_ = 1149.70; difference = -180.60, 95% CI [-398.15, 36.96], *t*(490.11) = -1.63, *p* = 0.104; *d* = -0.15, 95% CI [-0.32, 0.03]).

We conducted the exact same analysis performed on first fixation dwell times on the second fixation. The MAX*lmer* model fitting the dwell times distribution on the second fixation (*r*_c_ = .42, 95% CI [.38, .45], see Table 8SM in the MAXlmer models section) revealed an opposite pattern compared to the one observed on the first fixation. The MAXlmer model revealed:

1. a right-hemifield advantage, supported by longer dwell times for target on the right hemifield compared to targets the left hemifield (M_left_ = 1543.17, M_right_ = 1983.61; difference = -440.44, 95% CI [-578.36, -302.51], *t*(1194.14) = -6.27, *p* < .001; *d* = -0.36, 95% CI [-0.47, -0.24]).
2. a significant 3-ways interaction between Average Emotion Intensity × Target Position × Spatial Congruency interaction (*F*(1, 35.64) = 1.38, *p* = .246, η_p_^2^ = .042, 95% CI [.000, .222], which was consistent with a funnel pattern.
3. a main significant effect of Average Emotion Intensity (*F*(1, 104.19) = 17.07, *p* < .001, η_p_^2^ = .14, 95% CI [.04, .27]), which was consistent with a size effect. Participants dwell longer on a target within a globally positive than a negative pair (mean for globally positive pairs = 1942.72, mean of for globally negative pairs = 1650.26; difference = 292.46, 95% CI [431.51, 153.42], *t*(1197.45) = 4.13, *p* < .001; *d* = 0.24, 95% CI [0.12, 0.35]).

The results did not show any other significant main effects or interactions (*p >* .06)

The funnel pattern was characterized by a right-hemifield advantage (*c* = 235.68 ± 66.76, *t*(30) = -3.53, *p* = .001; *d* = -0.63, 95% CI [-1.03, -0.25]) combined with a happiness advantage (*K* = 64.08 ± 44.96, *t*(30) = -1.43, *p* = 0.164; *d* = -0.26, 95% CI [-0.62, 0.11]).The PAF and AAF resulted to be unbalanced in favor of AAF (*M_c_* = 0.22, 95% CI [0.07, 0.36], *t*(30) = 3.09, *p* = .004, *d* = 0.56, 95% CI [0.18, 0.95]). We corroborated the spatial congruity anisotropy by post-hoc analyses. In spatially congruent conditions, the funnel pattern was compatible with a happiness advantage (*k*_cong_ = 299.76 ± 105.97, *t*(30) = 2.83, *p* = .004; *d* = 0.51, 95% CI [0.13, 0.89]) and, in spatially incongruent conditions, the funnel pattern was compatible with an anger advantage (*k*_incong_ = -171.70 ± 41.56, *t*(30) = -4.13, *p* < .001; *d* = -0.74, 95% CI [-1.15, -0.34]). The difference between the *k*_cong_ vs *k*_incong_ (difference = -462.75, 95% CI [-684.23, -241.28], *t*(37.27) = -4.23, *p* < .001; *d* = -1.11, 95% CI [-1.65, -0.52]) was significant. In spatially congruent conditions, dwell times at the emotional rather than intermediate targets were larger in a globally positive pairs (M_angriest|intermediate_ = 1678.14, M_happiest|emotional_ = 2122.04, difference = -443.90, 95% CI [-730.63, -157.17], t(264.99) = -3.05, *p* = 0.003; *d* = -0.37, 95% CI [-0.60, -0.13]) compared to globally negative pairs (M_angriest|emotional_ = 1467.71, M_happiest|intermediate_ = 1787.99, difference = -320.28, 95% CI [-591.18, -49.38], *t*(310.65) = -2.33, *p* = 0.021; *d* = -0.26, 95% CI [-0.47, -0.04]). Conversely, in spatially incongruent pairs, dwell times for emotional targets rather than intermediate targets were larger in a globally negative pairs (M_angriest|emotional_ = 1962.04, M_happiest|intermediate_ = 1336.85, difference = 625.19, 95% CI [877.41, 372.97], *t*(334.71) = 4.88, *p* < .001; *d*  = 0.53, 95% CI [0.75, 0.31]) compared to globally positive pairs (M_angriest|intermediate_ = 2116.44, M_happiest|emotional_ = 1800.82, difference = 315.62, 95% CI [629.92, 1.31], *t*(183.47) = 1.98, *p* = .049; *d* = 0.25, 95% CI [0.50, 0.01]).

The final analysis aimed to test the accuracy of integrated SIA predictions on each fixation. This was done by remapping the individual values associated with the experimental factors (such as emotional intensity, spatial congruency, and target position) separately for the first and second fixation. The most parsimonious linear combination of SIA intensity components was applied to each individual's pattern of dwell times. More information on this analysis can be found in Data analysis subsection of the manuscript. SIA remapped values fully accounted for both the pattern of dwell times of the first (with empirically determined parameters *K* = 11.33, *c* = -23.41 and α = 0.4) and the second (with empirically determined parameters *K* = 64.08, *c* = 235.68 and α = 1.2) fixation. The remapped values were the only significant factor of the MAX*lmer* model in both the First fixation (*F*(1, 65.56) = 48.88, *p* < 0.001, η_p_^2^ = .43, 95% CI [.25, .57]) and the Second fixation (*F*(1, 220.42) = 6.09, *p* = .014, η_p_^2^ = .03, 95% CI [.01, .08]).

## MAXlmer models

**Table 5SM.** The summary table of the MAXlmer model including Average Emotion Intensity × Target Position × Spatial Congruency. The random structure was: Average Emotion Intensity × Target Position × Spatial Congruency | Subj.

|  | **Dwell times** | | |
| --- | --- | --- | --- |
| *Predictors* | *Estimates* | *CI* | *p* |
| (Intercept) | 1468.59 | 1252.30 – 1684.89 | **<0.001** |
| Target position [RIGHT] | -269.27 | -498.43 – -40.12 | **0.021** |
| Congruency [INCONG] | -320.26 | -515.48 – -125.04 | **0.001** |
| Average emotion intensity [50] | -53.01 | -238.44 – 132.42 | 0.575 |
| Target position [RIGHT] × Congruency [INCONG] | 619.47 | 308.88 – 930.06 | **<0.001** |
| Target position [RIGHT] × Average valF [50] | 697.01 | 423.87 – 970.16 | **<0.001** |
| Congruency [INCONG] × Average emotion intensity [50] | 816.55 | 543.81 – 1089.29 | **<0.001** |
| Target position [RIGHT] × Congruency [INCONG]) × Average emotion intensity [50] | -1487.98 | -1862.94 – -1113.03 | **<0.001** |

**Table 6SM.** The summary table of the MAXlmer model including Average Emotion Intensity × Target Position × Spatial Congruency × Fixation count. The random structure was: Average Emotion Intensity × Target Position × Spatial Congruency × Fixation count | Subj.

|  | **Dwell times** | | |
| --- | --- | --- | --- |
| *Predictors* | *Estimates* | *CI* | *p* |
| (Intercept) | 1393.95 | 1132.33 – 1655.58 | **<0.001** |
| Target position [RIGHT] | -694.56 | -966.85 – -422.26 | **<0.001** |
| Congruency [INCONG] | -424.70 | -681.62 – -167.79 | **0.001** |
| Average emotion intensity [50] | -227.90 | -449.24 – -6.57 | **0.044** |
| Fixation count [2] | 136.09 | -166.70 – 438.89 | 0.378 |
| Target position [RIGHT] × Congruency [INCONG] | 873.89 | 499.98 – 1247.80 | **<0.001** |
| Target position [RIGHT] × Average emotion intensity [50] | 1124.91 | 784.59 – 1465.22 | **<0.001** |
| Congruency [INCONG] × Average emotion intensity [50] | 1122.35 | 768.37 – 1476.34 | **<0.001** |
| Target position [RIGHT] × Fixation count [2] | 938.99 | 451.73 – 1426.25 | **<0.001** |
| Congruency [INCONG] × Fixation count [2] | 276.00 | -80.75 – 632.76 | 0.129 |
| Average emotion intensity [50] × Fixation count [2] | 463.69 | 96.01 – 831.38 | **0.013** |
| Target position [RIGHT] × Congruency [INCONG]) × Average emotion intensity [50] | -2240.27 | -2715.33 – -1765.21 | **<0.001** |
| Target position [RIGHT] × Congruency [INCONG]) × Fixation count [2] | -570.90 | -1052.86 – -88.95 | **0.020** |
| Target position [RIGHT] × Average emotion intensity [50] × Fixation count [2] | -1077.91 | -1639.99 – -515.83 | **<0.001** |
| Congruency [INCONG] × Average emotion intensity [50] × Fixation count [2] | -989.00 | -1568.65 – -409.34 | **0.001** |
| Target position [RIGHT] × Congruency [INCONG] × Average emotion intensity [50] × Fixation count [2] | 1996.78 | 1240.26 – 2753.29 | **<0.001** |

**Table 7SM.** The summary table of the MAXlmer model performed on the first fixation and including Average Emotion Intensity × Target Position × Spatial Congruency. The random structure was: Average Emotion Intensity × Target Position × Spatial Congruency| Subj.

|  | **Dwell time** | | |
| --- | --- | --- | --- |
| *Predictors* | *Estimates* | *CI* | *p* |
| (Intercept) | 1393.41 | 1133.13 – 1653.68 | **<0.001** |
| Target position [RIGHT] | -694.92 | -961.41 – -428.43 | **<0.001** |
| Congruency [INCONG] | -425.36 | -669.68 – -181.04 | **0.001** |
| Average emotion intensity [50] | -227.10 | -442.64 – -11.55 | **0.039** |
| Target position [RIGHT] × Congruency [INCONG] | 875.75 | 516.35 – 1235.16 | **<0.001** |
| Target position [RIGHT] × Average emotion intensity [50] | 1123.06 | 784.05 – 1462.07 | **<0.001** |
| Congruency [INCONG] × Average emotion intensity [50] | 1123.51 | 756.23 – 1490.79 | **<0.001** |
| Target position [RIGHT] × Congruency [INCONG] × Average emotion intensity [50] | -2240.75 | -2690.01 – -1791.48 | **<0.001** |

**Table 8SM.** The summary table of the MAXlmer model performed on the second fixation and including Average Emotion Intensity × Target Position × Spatial Congruency. The random structure was: Average Emotion Intensity × Target Position × Spatial Congruency| Subj.

|  | **Dwell times** | | |
| --- | --- | --- | --- |
| *Predictors* | *Estimates* | *CI* | *p* |
| (Intercept) | 1502.12 | 1254.31 – 1749.92 | **<0.001** |
| Target position [RIGHT] | 261.21 | -130.79 – 653.21 | 0.191 |
| Congruency [INCONG] | -143.80 | -406.68 – 119.08 | 0.283 |
| Average emotion intensity [50] | 237.27 | -40.32 – 514.87 | 0.094 |
| Target position [RIGHT] × Congruency [INCONG] | 326.38 | -80.42 – 733.19 | 0.116 |
| Target position [RIGHT] × Average emotion intensity [50] | 60.54 | -352.74 – 473.82 | 0.774 |
| Congruency [INCONG] × Average emotion intensity [50] | 199.83 | -225.95 – 625.61 | 0.357 |
| (Target position [RIGHT] × Congruency [INCONG]) × Average emotion intensity [50] | -347.53 | -926.68 – 231.61 | 0.239 |

# **Analysis of Saccades latency**

## Descriptive statistics

The average latency with which participants performed the first saccade to orient attention towards one of the two faces were in the domain of 'fast regular' saccades being of about 239.01 ms (min-max range = 3-2497, SD = 135.59) (Fischer and Boch, 1983; Kingstone & Klein, 1993; Wenban-Smith & Findlay, 1991).

## Analysis of the type of saccade

Participants made more saccades between faces (moving from one face to the other, from the first to the second fixation) than within faces (0.55 vs. 0.45, 95% CI [.02, .16], *t*(64) = 2.637, *p* = 0.01) Among the between faces saccades, the proportion of corrective saccades (i.e., moving from the wrong face to the target face) was about 0.72 (SD= 0.45).

## MAXlmer model

**Table 9SM.** The summary table of the MAXlmer model including Average Emotion Intensity × Target Position × Spatial Congruency × Fixation accuracy. The random structure was: Average Emotion Intensity × Target Position × Spatial Congruency × Fixation accuracy | Subj.

|  | **Saccade latency** | | |
| --- | --- | --- | --- |
| *Predictors* | *Estimates* | *CI* | *p* |
| (Intercept) | 404.13 | 350.32 – 457.94 | **<0.001** |
| Target position [RIGHT] | -73.64 | -152.94 – 5.65 | 0.069 |
| Congruency [INCONG] | -8.29 | -87.90 – 71.31 | 0.838 |
| Average emotion intensity [50] | -90.22 | -172.41 – -8.03 | **0.031** |
| Fixation accuracy [1] | 138.25 | 52.53 – 223.98 | **0.002** |
| Target position [RIGHT] × Congruency [INCONG] | -22.90 | -134.44 – 88.65 | 0.687 |
| Target position [RIGHT] × Average valF [50] | 78.79 | -37.29 – 194.86 | 0.183 |
| Congruency [INCONG] × Average emotion intensity [50] | 8.33 | -113.00 – 129.66 | 0.893 |
| Target position [RIGHT] × Fixation accuracy [1] | 39.42 | -82.65 – 161.50 | 0.527 |
| Congruency [INCONG] × Fixation accuracy [1] | -56.86 | -157.60 – 43.89 | 0.269 |
| Average emotion intensity [50] × Fixation accuracy [1] | 62.58 | -37.46 – 162.62 | 0.220 |
| (Target position [RIGHT] × Congruency [INCONG]) × Average emotion intensity [50] | 10.30 | -156.70 – 177.31 | 0.904 |
| (Target position [RIGHT] × Congruency [INCONG]) × Fixation accuracy | 144.48 | 7.25 – 281.72 | **0.039** |
| (Target position [RIGHT] × Average valF [50]) × Fixation accuracy [1] | -19.63 | -159.03 – 119.78 | 0.783 |
| (Congruency [INCONG] × Average emotion intensity [50]) × Fixation accuracy [1] | 18.59 | -130.56 – 167.73 | 0.807 |
| (Target position [RIGHT] × Congruency [INCONG] × Average emotion intensity [50]) × Fixation accuracy [1] | -110.81 | -316.11 – 94.48 | 0.290 |
